# Strong Bases and beyond: The Prominent Contribution of Neutral Push–Pull Organic Molecules towards Superbases in the Gas Phase

**DOI:** 10.3390/ijms25115591

**Published:** 2024-05-21

**Authors:** Ewa Daniela Raczyńska, Jean-François Gal, Pierre-Charles Maria

**Affiliations:** 1 Department of Chemistry, Warsaw University of Life Sciences (SGGW), ul. Nowoursynowska 159c, 02-776 Warsaw, Poland; 2 Institut de Chimie de Nice, UMR 7272, Université Côte d’Azur, Parc Valrose, 06108 Nice, France; pierre-charles.maria@univ-cotedazur.fr

**Keywords:** N-sp^3^, N-sp^2^ and N-sp containing organic nitrogen superbases, gas-phase proton basicity, isomeric phenomena, substituent effects, intramolecular interaction effects, PA/GB measurements and calculations, PA/GB scales

## Abstract

In this review, the principles of gas-phase proton basicity measurements and theoretical calculations are recalled as a reminder of how the basicity PA/GB scale, based on Brønsted–Lowry theory, was constructed in the gas-phase (PA—proton affinity and/or GB—gas-phase basicity in the enthalpy and Gibbs energy scale, respectively). The origins of exceptionally strong gas-phase basicity of some organic nitrogen bases containing N-sp^3^ (amines), N-sp^2^ (imines, amidines, guanidines, polyguanides, phosphazenes), and N-sp (nitriles) are rationalized. In particular, the role of push–pull nitrogen bases in the development of the gas-phase basicity in the superbasicity region is emphasized. Some reasons for the difficulties in measurements for poly-functional nitrogen bases are highlighted. Various structural phenomena being in relation with gas-phase acid–base equilibria that should be considered in quantum-chemical calculations of PA/GB parameters are discussed. The preparation methods for strong organic push–pull bases containing a N-sp^2^ site of protonation are briefly reviewed. Finally, recent trends in research on neutral organic superbases, leaning toward catalytic and other remarkable applications, are underlined.

## 1. Introduction

The realm of very strong bases has known an extraordinary development in the last decades—in particular, their experimental and theoretical characterization and uses. According to Trofimov and Schmidt [1], the concept of superbasic media appeared in 1977, although the term “superbase(s)” occurred earlier (see, for example, Ref. [2]). In this article, the “superbase(s)” terminology is relative to mixtures of metal salts (usually of alkali metals) of weak acids, or hydroxides, or their mixtures in given solvents. Caubère, in his review article published in 1993, used the term “super bases” for such composite media [3]. For gaseous species, the term “superbase(s)” was also employed early. For example, in 1977, Beauchamp et al. applied the name “superbase” to the metal atom uranium [4]. Taft and our group from Nice used the term “superbases” in 1986 for amino-enimines containing the conjugated moiety R’_2_N–C=C–C=NR partially closed in the cyclohexene ring [5].

In the general context of chemistry, the precise signification of superbasicity remains elusive. In the latest version of the IUPAC (International Union of Pure and Applied Chemistry) glossary of physical organic chemistry, the definition of superbases is cursory: “compound having a very high basicity” [6]. The definition includes two examples of alkali metal salts, or their mixture, and phosphazenes. An ambiguity still remains in this 2022 glossary, superbases including inorganic salts and neutral purely organic bases. The entry of the glossary cites one reference to purely organic bases. In fact, for some time, the term superbases implied neutral organic bases [7].

Owing to the theme of this special issue, and given the ambiguous usage of superbase and superbasicity, we specify hereafter that the term “superbase(s)” (or “superbasicity”) will be used in relation to neutral (uncharged) very strong organic bases. Such “electron abundant” (electron rich) neutral organic compounds present several advantages over inorganic or charged bases, especially in catalysis [8].

The thermochemical gas-phase proton-basicity parameters (GB—gas-phase basicity and PA—proton affinity [9], definitions of which are recalled below) can be obtained experimentally by different mass spectrometry techniques or estimated theoretically by quantum chemistry calculations. In both experimental and theoretical procedures, the proton, the neutral base, and its protonated form (ionic conjugate acid) are in the gas phase but are usually treated differently. In mass spectrometers, a mixture of neutral and protonated species is in a reduced pressure environment at ca. 300–600 K, whereas in calculations, isolated individual species are separately considered at 298 K and 1 atm or 1 bar.

The quantitative comparison of base strength in the gas phase becomes customary, principally because a large experimental GB and PA data base is now freely available in the NIST WebBook [10]. Another advantage of using these measures of the proton basicity is that the absence of solvation on the species, involved in protonation–deprotonation equilibria in the gas phase and facilitates quantum chemistry calculations of the corresponding thermochemistry, resulting in useful assessment of theory and experiments.

In fact, the thermochemistry of Brønsted acidity and basicity in vacuo renders possible the dissection of various solvation effects. These aspects resulting from the comparison of the proton transfer in the gas phase and in solution appear in many works that will be cited in this review. On the other hand, the absence of solvation gives insight on intrinsic structural (internal) substituent effects using carefully chosen model systems. In more complex isomeric and polyfunctional organic molecules, specific effects can be identified. For these reasons, the gas-phase data focused the attention of many researchers as soon as they were sufficiently accurate and widely available. The resulting analyses of internal effects help in designing new structures of compounds displaying extreme properties. They are also useful for the explanation of mechanisms of various reactions in which organic base plays the role of substrate, nucleophile, or catalyst [7].

In this review, first the terminology of super systems, especially superbases, is clarified. The basics of thermochemistry of gas-phase proton basicity, the experimental aspects of GB and PA determinations, and the theoretical approaches in GB and PA estimations are also recalled. Then, experimental basicities—and in some cases, also calculated basicity parameters for selected organic nitrogen strong bases with a N-sp^3^, N-sp^2^, or N-sp site of protonation—are briefly reviewed. All literature reports in this field, including our pioneering works, reporting majority of experimental PA/GB data for strong organic nitrogen bases, and those of other chemists from the USA, Canada, France, Spain, Estonia, Japan, Croatia, the Czech Republic, Germany, New Zealand, and Iran are considered and discussed. The research progress is displayed in both PA and/or GB measurements, showing the extension of the experimental PA/GB scale toward push–pull and/or polydentate organic nitrogen superbases, including natural products such as aminoacids and bioamines, containing the additional amino, amidino, or guanidino group. Particular attention is drawn to the most important structural effects that affect base strength in the gas phase. Finally, some preparative methods employed for very strong push–pull organic nitrogen bases containing a N-sp^2^ protonation site—such as amidines, guanidines, biguanides, and phosphazenes that can serve as a guide for syntheses of new series of superbases—are summarized. In the last part, the evolution of the knowledge in the field of strong organic nitrogen bases and the diversity of their significant applications are presented.

## 2. When Do Exceptionally Strong Organic Bases Become Superbases?

In chemistry, various species possessing exceptional properties are very often dubbed “super”, and the following terms can be found in the literature: superatoms, superalkalis, superhalogens, supermolecules, superacids, superbases, etc. Superatoms are clusters of atoms that mimic properties of classical atoms. In particular, superalkalis and superhalogens have exceptional properties like alkali metals and halogens, respectively [11]. Supermolecules refer to non-covalently bonded adducts of molecules that preserve their own properties [12]. Superacids are usually defined as species of stronger acidity than 100 wt.% sulfuric acid [13,14].

According to the IUPAC terminology [6], superbases (organic, but also organometallic compounds) exhibit very high basicities, such as lithium di-isopropylnitride (LiNPr^i^_2_). However, many chemists use the term “superbases” for purely organic Brønsted bases, stronger in solution than proton sponge, 1,8-bis(dimethylamino)naphthalene (**DMAN**, p*K*_a_ 12.34 in water [15] and 18.6 in acetonitrile [16]).

**Figure 1:**
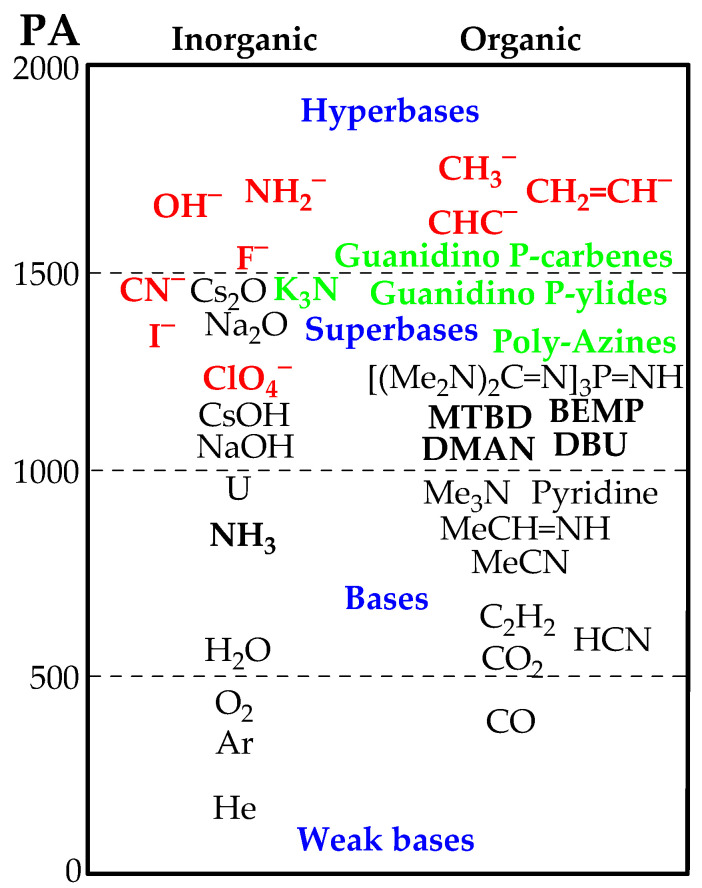
General PA scale (in kJ mol^−1^) for inorganic and organic bases. Formulae (or names) of neutral derivatives, written in black and green, refer to those with measured and calculated PAs, respectively. Formulae of some anionic bases (conjugate forms of weak neutral acids) are shown in red.

In the gas phase, very strong bases have extremely high PAs. If neutral inorganic species are considered, some of them display exceptional affinity to protons and can be classified as superbases. For example, some atomic elements (Y, Lu, U, and La; PA 967, 992, 995, and 1013 kJ mol^−1^, respectively [9]), alkali metal molecules (Li_2_ and Na_2_; PA 1162 and 1147 kJ mol^−1^, respectively [9]), alkali metal hydroxides (LiOH, NaOH, KOH, and CsOH; PA 1000–1118 kJ mol^−1^ [9]), alkaline earth oxides, and alkali metal oxides (MgO, CaO, BaO, SrO. Li_2_O, Na_2_O, K_2_O, Cs_2_O; PA 988–1443 kJ mol^−1^ [9]) have PAs close to or higher than 1000 kJ mol^−1^ (see Figure 1, data taken from Refs. [9,10,17,18,19]).

When our pioneering experimental investigations were started in the 1990s for gaseous organic nitrogen compounds of exceptional Brønsted basicity containing the amidino or guanidino group, we proposed at first that bases exhibiting a PA > 1000 kJ mol^−1^ will be stamped as superbases [20,21]. The PA values of strong inorganic hydroxides (MOH, M alkali metal cation) start at 1000 kJ mol^−1^, and thus, Brønsted neutral organic bases with PAs comparable to those of MOH can be considered as superbases. A few years later, we proposed **DMAN** (GB 995.8 kJ mol^−1^ and PA 1028.2 kJ mol^−1^ [9]) as the cornerstone for superbases [22]. Our cornerstone for superbasicity has been accepted by many other scientists in the field of gas-phase physical organic chemistry. This convention, based on the so called “proton sponge” **DMAN**, is historically defendable, and seems better than an arbitrary threshold, subject to experimental or theoretical challenge. As Brønsted basicity is linked to a Gibbs energy in the IUPAC definition, this may be more consistent for referring to the GB (**DMAN**) in the gas phase as well as to the p*K*_a_(**DMAN**) in solution.

Considering the current experimental PA scale for neutral bases [9,18], derivatives exhibiting a PA beyond that of the inorganic base Cs_2_O (PA 1443 kJ mol^−1^ [9]) can deserve the name of hyperbases in the gas phase (Figure 1). Note that organic neutral superbases in the experimental PA scale have not yet achieved a PA of 1200 kJ mol^−1^ [18,20,21,22,23,24,25,26,27,28]. The terms “hyperbases”, “hyperbasicity”, and “hyper strong” were applied to calculated PAs of neutral inorganic (e.g., tripotassium nitride, K_3_N, PA 1458 kJ mol^−1^ [17]) and organic bases (e.g., phosphazeno azacalix[3](2,6)pyridine, PA 1316 kJ mol^−1^ [29], some polycyclic diazines, PA ≥ 1300 kJ mol^−1^ [30], guanidino phosphorus ylides and guanidino phosphorus carbenes PA ≥ 1400 kJ mol^−1^ [19]). Finally, if taking into account anionic bases (conjugate bases of Brønsted neutral inorganic and organic acids), the strongest anions among them can be also considered as hyperbases. This term particularly concerns the conjugate bases of exceptionally weak acids. For example, the following inorganic and organic anions can be considered as hyperbases in the gas phase: H_3_C–CH_2_^−^, CH_3_^−^, H_2_C=CH^−^, NH_2_^−^, H^−^, OH^−^, CH_3_O^−^, HC≡C^−^, and F^−^. Their PAs are between 1500 and 1800 kJ mol^−1^ [10].

## 3. Thermodynamic Acid–Base Parameters in the Gas Phase

According to the Brønsted–Lowry theory the species able to lose a proton is called an acid, and that able to capture a proton is called a base [31,32]. In the gas phase, we can write a formal Equilibrium (1) between the conjugated pair of acid and base species. There is no solvent nor other species participating in this deprotonation-protonation reaction that defines acid-base properties. Equilibrium (1) relates two types of conjugate pairs: (i) neutral acid and its anionic base (AH ⇄ A^−^ + H^+^) as well as (ii) cationic acid and its neutral base (BH^+^ ⇄ B + H^+^) [9,10].

Acid ⇄ Base + H^+^ (1)

Considering the basicity of the neutral base B, BH^+^ is its conjugate acid. The basicity parameters for B can be defined from the thermodynamics of Equilibrium (1). Proton affinity (PA, Equation (2)) for B is defined as the enthalpy change of deprotonation reaction (1) or, conversely, as the negative of the enthalpy change of the reverse protonation reaction [9]. If the Gibbs energy of (1) is considered, this thermodynamic parameter is called gas-phase basicity (GB, Equation (3)). Very often, the PA and GB values compiled in the NIST Chemistry WebBook correspond to 298 K [10]. The literature also provides thermodynamic acidity parameters, such as the deprotonation enthalpy (DE = Δ_acid_*H*) and gas-phase acidity (GA = Δ_acid_*G*) for an acid AH that are equal to the PA and GB of its conjugate base A^−^, respectively. The use of different abbreviations has only a historical connotation. In Equation (3), Δ*S* is the entropy of deprotonation.

PA = Δ*H*(Acid → Base + H^+^) = −Δ*H*(Base + H^+^ → Acid) (2)

GB = Δ*G*(Acid → Base + H^+^) = −Δ*G*(Base + H^+^ → Acid) = PA − *T*Δ*S* (3)

The thermodynamic parameters of the acid–base Equilibrium (1) in the gas phase, PA and GB, correspond to absolute (intrinsic) basicity parameters. They depend only on the structure of the investigated species (the conjugated acid–base pair), i.e., its protonation-deprotonation site and all internal effects. PA and GB increase when the strength of the base increases or when the strength of the conjugate acid decreases. For most bases, Equilibrium (1) can be considered as a quasi-isentropic reaction with the entropy term (*T*Δ*S*) taken as equal to 32.5 kJ mol^−1^, i.e., only the entropy of the free proton is considered in Equation (3). However, when a base and its conjugate acid after protonation exhibit a symmetry or conformation change, the entropy term may be different. This question was discussed in our tutorial review on the thermochemistry of the proton-transfer reaction [33].

## 4. Experimental Methods of PA/GB Determinations: Superbase History and Recent Advances

Several mass-spectrometric methods can be used for the determination of gas-phase basicities, based on the observation of proton transfer in a low-pressure environment [33,34,35]. Among them, the ion cyclotron resonance (ICR), mostly used in its Fourier transform mode (FT-ICR) [36], and the Cooks’ kinetic method [37,38] are essentially retained here. The thermokinetic (earlier called “bracketing” in its semi-quantitative form) method developed by Bouchoux [39] was not used for the extension of the gas-phase basicity scale, as it is essentially an interpolation method, i.e., it requires known weaker and stronger bases to place an unknown base in the basicity scale. The so-called high pressure mass spectrometry (HPMS, in fact working at a few mbar of a bath gas containing a very small partial pressure of the reactants) was also used for basicity determination for a series of “proton sponges” only once in the context of superbases [40]. Consequently, the use of ICR techniques for the development of the basicity scale above PA = 1000 kJ mol^−1^ has been reminded here.

The heart of the ICR or FT-ICR technique is to trap ions at very low pressures in combined magnetic/electrostatic fields, for relatively long times, i.e., from several seconds to minutes. Under these conditions, the trapped ions may be subjected to reactions with neutral gases (ion/molecule reactions). The equilibria and kinetics of proton transfer reaction were frequently studied by ICR techniques. In the present review, we concentrate on the thermodynamics of proton transfer between neutral bases and their corresponding protonated species.

In FT-ICR mass spectrometry, the proton-transfer Equilibrium (4) can be studied between two bases, B and B′. The basicity of B is unknown and that of B′ (reference base) is already measured. The difference in gas-phase basicities between B′ and B (relative basicity in term of Gibbs energy), ΔGB = GB(B′) − GB(B) can be established for (4) from the equilibrium constant *K* (Equation (5)). When equilibrium is achieved, the unitless *K* value is obtained from the ratio of the partial pressures (*p*) of the two bases, B and B′, and the relative concentration (actually relative intensity (*I*) in the mass spectrum) of the two ions, BH^+^ and B′H^+^ (Equation (6)). The conditions for measurements—especially of pressures, attainment of equilibrium, and the problem of the temperature of the ICR cell—were discussed in Refs. [33,35].

B + B′H^+^ ⇄ BH^+^ + B′ (4)

ΔGB = −*RT* ln*K* (5)

*K* = [*I*(BH^+^) *p*(B′)]/[*p*(B) *I*(B′H^+^)] (6)

Early measurements using ICR, with which the spectra recording were rather long, probably missed the attainment of the equilibrium state in a number of cases. With FT-ICR instruments, the spectrum is taken within seconds, and Equilibrium (4) may be reached in reasonable lengths of time. In a detailed description of the experimental set-up and conditions, it is stated that in some cases a minimum equilibration time of 800 s is needed to reach equilibrium of proton exchange [25]. There are two other tests that can be carried out to check the consistency of the *K* value: (i) selective ejection of one of the ions involved in Equilibrium (4) and following the return to the equilibrium and (ii) variation of the relative partial pressures of the bases.

**Figure 2:**
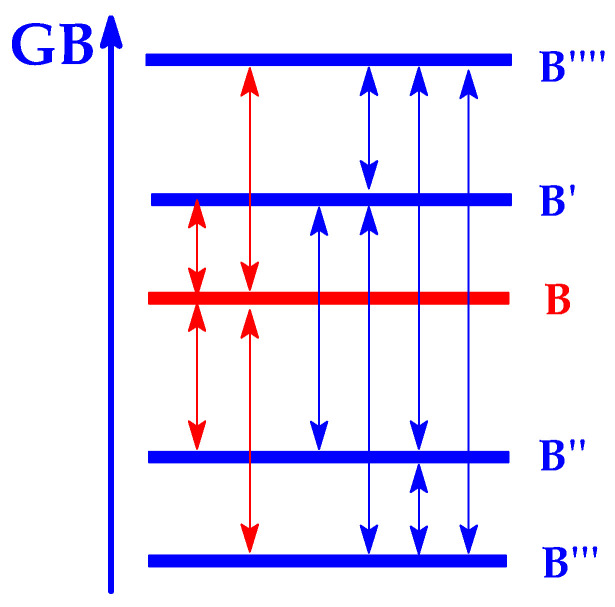
Relative basicities in the GB scale between base B of unknown basicity and reference bases B′, B″, B‴, and B′′′′ (red arrows); the quality of the overlap between the relative GBs (blue arrows) can be assessed using the equilibrium method.

The difficulty of partial pressure measurements of the superbases and of the temperature, at which the equilibrium constant is determined, are also detailed in Ref. [25]. Once the ΔGB value between the two bases, B and B′, has been established, another measurement between B and a third base B″ can be taken, then between B and B‴, between B and B′′′′, etc. A further verification of the self-consistency of the scale is to check the quality of the overlap between the ΔGB values by performing measurement between various reference bases, e.g., B′ and B″, B′ and B‴, B′ and B′′′′, B″ and B‴, B″ and B′′′′, etc. [18,24,25], see Figure 2. If this figure is supposed to be the upper part of the GB scale, bases exhibiting GBs higher than GB(B′′′′) can be used to extend the scale step by step.

The advances in the domain of gas-phase basicity of superbases are intimately linked to the developments of the entire gas-phase basicity scale. After the era of qualitative ranking of bases using more or less modified standard mass spectrometers (see for example Ref. [41]), the design of specific instruments allowed more quantitative evaluation of gas-phase basicities. As most gas-phase basicity parameters (PA and GB) originate from relative measurements, assignments of some absolute PA and GB values were necessary to get a reliable basicity scale on the entire range. The extension of PA and GB scales in the high-basicity range has long been fraught with uncertainties. In the current basicity scale, ammonia (NH_3_) is the strongest base serving as a primary standard to anchoring for the higher part of the scale [9]. In 1984, the PA value of ammonia was still uncertain by ±12 kJ mol^−1^ [42], but fortunately, the PA value selected in 1984 (853.5 kJ mol^−1^) was confirmed on the basis of accurate ab initio calculations (853.6 kJ mol^−1^) [43]. In 1998, Hunter and Lias [9] added more basic secondary standards.

Most measurements were performed by proton-transfer equilibria, leading to relative Gibbs energies, Equation (5), which requires the knowledge of the temperature. In early ICR measurements, the real temperature of the cell was rarely mentioned and implicitly taken as 298 K (see for example [44,45,46]). When we became aware of this difficulty, we tried to be more specific in our articles (see for example Ref. [47]). The problem of temperature measurement and the corresponding corrections were discussed in the 1998 compilations of Hunter and Lias [9]. The early ICR [48,49] and HPMS [40] measurements on superbases were corrected accordingly in Ref. [9].

The relative ΔGBs can be linked, or “anchored”, to one or several absolute GB values, which rely in fact on absolute PA values. For establishing the correspondence between the enthalpy value (PA), and the Gibbs energy (GB), the entropy must be evaluated [9,33,35]. All values reported hereafter refer to the standard temperature of 298 K. The primary anchor point of the current gas-phase basicity scale [10] remains ammonia (NH_3_, GB 819.0 kJ mol^−1^; PA 853.6 kJ mol^−1^), and secondary evaluated relative basicities are also available [9]. The strongest base, used as a secondary anchor point, is trimethylamine (Me_3_N, GB 918.1 kJ mol^−1^; PA 948.9 kJ mol^−1^). It is worth noting that the accuracy of values listed in the NIST database are dependent on the anchoring process and on the interconnection of the individual measurements. When comparing bases, it can be stated that basicity differences (relative basicities) of compounds relatively close in the PA/GB scales are more accurate that the absolute basicities considered separately.

The experimental PA values close to or larger than 1000 kJ mol^−1^, measured by ICR [48,49] or HPMS techniques [40], were already reported in the 1970s. The gas-phase basicity of diamines studied in these works was enhanced by internal hydrogen bonding, corresponding to a “proton chelation”, thanks to the absence of solvation of the amino and ammonium groups. These data were reported in subsequent reviews [42,50]. At those times, the largest gas-phase basicities of monofunctional organic nitrogen bases, for which stabilization by proton chelation is absent, were documented for tri-n-butylamine (n-Bu_3_N), *N*,*N-*dimethyl-4-aminopyridine (4-Me_2_N-C_5_H_4_N, **DMAP**), N^1^,N^1^,N^3^,N^3^-tetramethylguanidine ((Me_2_N)_2_C=NH, **TMG**), and **DMAN** [5,40,42,50]. Their PA values, evaluated by Hunter and Lias in 1998 [9], are as follows: 998.5, 997.6, 1031.6, and 1028.2 kJ mol^−1^, respectively.

In the 1990s, it was observed in the gas phase GB measurements that the basicity of imino nitrogen in N^1^,N^1^-dimethylformamidines (Me_2_NCH=NR) is enhanced by a push–pull effect. For the series of phenyl derivatives, PA values close to that of tripropylamine (n-Pr_3_N) were measured by ICR [51,52]. For extended series of alkyl and heteroalkyl derivatives, the measured PAs are close to or higher than that of n-Bu_3_N [53,54]. As stated above, the strongest reference bases that can be used as an anchor point in the gas-phase basicity scale was Me_3_N. The GB range for other series of push–pull derivatives (R”_2_NCR’=NR and (R’_2_N)_2_C=NR) was too high to be anchored to this compound. For this reason, **TMG** was chosen as the first anchor-point for our pioneering GB measurements of superbases [20,21,22,23,55]. This simple compound is relatively volatile and stable for gas-phase measurements, in particular by FT-ICR. As a commercial product, it is also easily accessible. Its GB value, evaluated in 1998 by Hunter and Lias (997.4 kJ mol^−1^ [9]), is strongly connected to the other basicities in the scale, and is close to those for investigated push–pull alkyl- and heteroalkyl-imines (amidines and guanidines). Our works contributed to the step-by-step extension of the GB scale, corresponding to PAs up to ca. 1100 kJ mol^−1^ [18]. The evaluated PA value for this anchor point (1031.6 kJ mol^−1^ [9]) was confirmed in 2016 by Bouchoux and Eckert-Maksić [56], who calculated the PA of **TMG** at the exceptionally high G4MP2 level and found a very close theoretical PA value (1031.3 kJ mol^−1^).

The Koppel and Leito group from University of Tartu (Estonia) in cooperation with Mishima from Kyushu University, Fukuoka (Japan) extended the PA/GB scale for superbases, using bicyclic guanidine, 7-methyl-1,5,7-triazabicyclo[4.4.0]dec-5-ene (**MTBD**), as the next anchor point for equilibrium measurements for series of very strong phosphazenes [24,25,57]. This bicyclic guanidine possesses qualities similar to those of **TMG**, i.e., volatility, stability, and availability. **MTBD** was also used as the anchor point by Glasovac et al. (Ruđer Bošković Institute, Zagreb, Croatia), who investigated series of polyfunctional superbases, guanidines, and biguanides [26,27,28]. The two groups used the PA/GB values of **MTBD** (1062.7/1030.2 kJ mol^−1^) evaluated by Hunter and Lias in 1998 on the basis of our preliminary experiments published in the 1990s and based on **TMG** as the anchor point. However, according to our last re-evaluation of basicity parameters for superbases [18], the PA/GB values of **MTBD** should be slightly higher (1065.7/1033.2 kJ mol^−1^). Unfortunately, there are no high-level computational studies of PA/GB for **MTBD** to be compared with experiment data.

## 5. Procedures for PA/GB Theoretical Estimations

As early as the Brønsted–Lowry theory for acids and bases has been proposed in the literature, quantum theoretical investigations were performed for the simplest neutral bases, including the nitrogen base NH_3_ [58], one of the primary anchor-points in the experimental PA/GB scale [9,42]. With development of quantum chemical methods and computational techniques, the level of computations increased from semi-empirical to more accurate DFT and ab initio levels (HF, MP*n*, CCSD(T), QCISD(T), W1, W2, and G*n*). The last compilation of experimental PA/GB data, published by Hunter and Lias in 1998 [9], was backed by the G2 results of Smith and Radom published in 1993 for computed PA values of 31 molecules, including NH_3_ [43]. For exceptionally strong nitrogen bases, quantum chemical calculations have been carried out in numerous laboratories in the world since the 1990s. For more details on some considered structures and estimated PAs, see our previous review article for strong push–pull organic nitrogen bases [18]. For theoretical PA estimations, chemists used various levels of theory. For example, Koppel, Leito, et al. mainly used the DFT methods [17,19,59]. Maksić et al. applied both the DFT and MP*n* methods [60], while Bouchoux employed G*n* theories for amino acids (including the superbase arginine) [61] and for polyfunctional N-sp^2^ containing bases (including amidines and guanidines) [56].

For each base site in inorganic or organic superbases, thermodynamic gas-phase basicity parameters (PA and GB) can be estimated for Equilibrium (1) using DFT or ab initio methods using two different procedures. In one of them (presently the most frequently used), Equations (7) and (8) can be applied, respectively. In these equations, the calculated enthalpies (*H*) and Gibbs energies (*G*) of the conjugate pairs (base and acid forms) correspond to 298 K. Considering the proton as a quasi-classical particle, the following values can be used: *H*(H^+^) = 6.2 kJ mol^−1^ and *G*(H^+^) = − 26.3 kJ mol^−1^ at 298 K [62,63]. The proton enthalpy corresponds to the translational energy term 3/2*RT* and the work term *pV* (equal to *RT* for an ideal gas), *H*(H^+^) = *E* +*pV* = 3/2*RT* + *RT* = 5/2*RT*. The proton Gibbs energy can be found from the translational entropy *S*(H^+^) = 108.95 J mol^−1^ K^−1^ and the relation *G*(H^+^) = *H*(H^+^) − *TS*(H^+^).

PA(Base) = *H*(Base) + *H*(H^+^) − *H*(conjugate Acid) (7)

GB(Base) = *G*(Base) + *G*(H^+^) − *G*(conjugate Acid) (8)

In the other procedure, PA can be calculated according to Equation (9) from the standard enthalpies of formation (Δ_f_*H*) of the considered base, its conjugate acid, and its proton. This procedure has been applied in theoretical PA predictions by Bouchoux and co-workers, who employed the so-called “ion convention” and Δ_f_*H*(H^+^) = 1530 kJ mol^−1^ [9] (see series of review articles on gas-phase basicity of polyfunctional molecules, for example part 1 on theory and methods [35]). The GB values can be estimated for selected bases using Equation (10), in which the entropy change of Equilibrium (1) can be calculated from Equation (11). All calculated parameters refer to 298 K.

PA(Base) = Δ_f_*H*(Base) + Δ_f_*H*(H^+^) − Δ_f_*H*(conjugate Acid) (9)

GB(Base) = PA(Base) − *T*Δ*S* (10)

Δ*S = S*(Base) + *S*(H^+^) − *S*(conjugate Acid) (11)

It should be mentioned here that in several Gaussian documents (see for example Ref. [64]) containing exercises for calculations of different geometric and energetic parameters, there are examples of proton affinity calculations. The procedure of PA calculation, given in this manual, corresponds to 0 K, for which *E*(H^+^) = 0 and the proposed equation for PA reduces to: PA = *E*(Base) − *E*(conjugate Acid) with ZPE but without changes in thermal corrections (vibrational, rotational, and translational). This rough approximation of experimental data obtained at room temperature was frequently used by many chemists in the end of the 20th century and even sometimes in the last 20 years. However, such calculations correspond to the energy of protonation *E*_prot_. This energetic parameter calculated for 0 K cannot be considered as PA defined in Equation (7) for 298 K and cannot be compared to experimental PA that also refers to 298 K. The work of Bartmess published in 1994 [62], and next that of Fifen et al. published in 2014 [63], clarified both the thermochemistry of the proton in the gas phase and PA calculations using quantum chemical methods for 298 K.

## 6. Theoretical Treatment of Isomeric Bases

Employing Gaussian (or other) series of programs implemented on a suitable personal computer—or better, using computing facilities provided by a large computer center—one can carry out quantum chemical calculations for any strong organic nitrogen acid–base conjugate pair. However, it should always be verified if isomeric phenomena do not operate in both the neutral and protonated forms. When isomers (rotamers and/or tautomers) are not possible for investigated derivative, calculations can be reduced to two structures (neutral and protonated), and PA or GB parameters can be estimated according to Equations (7) and (8) or (9)–(11).

A different situation takes place for acyclic or mixed acyclic–cyclic molecules with flexible structure. In such cases, rotational isomerism about all single bonds should be considered for neutral base and protonated (conjugate acid) forms. A similar situation occurs for cyclic tautomeric systems, for which all reasonable tautomeric structures should be analyzed for the neutral and protonated forms. Situation is more complicated for polyfunctional bases with flexible structure, for which all possible sites of protonation and all possible conformations for neutral and protonated forms should additionally be considered. Additional complications take place for tautomeric systems with flexible structures that are the most complex cases. For such compounds, the different combinations of protonation sites and tautomers-rotamers should be analyzed. Extensive prototropic equilibria and a large number of single bonds in the system induce a large number of possible isomers (tautomers-rotamers) that should be considered for the neutral and protonated forms. Calculations require excellent knowledge of isomeric phenomena, particular concentration, and extreme patience from researchers.

**Figure 3:**
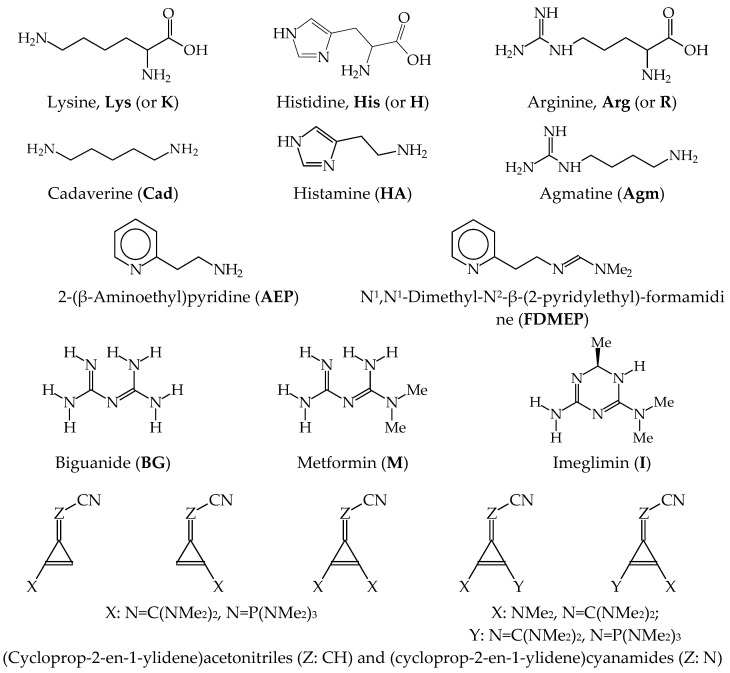
Examples of structures with flexible conformation and/or prototropy: α-amino acids (lysine, histidine, and arginine), their bioamines (cadaverine, histamine, and agmatine), aminazine (2-(β-aminethyl)-pyridine}, azino-amidine {2-(dimethylformamidinethyl)-pyridine), biguanides (biguanide, metformin, and imeglimin), and nitriles (series of mono- and di-substituted cycloprop-2-en-1-ylidene)acetonitriles and (cycloprop-2-en-1-ylidene)cyanamides).

Figure 3 presents some examples of polyfunctional organic nitrogen compounds with flexible structures that display strong basicity in the gas phase: α-amino acids (lysine, histidine, and arginine), the corresponding bioamines (cadaverine, histamine, and agmatine), amino-azine (2-(β-aminoethyl)pyridine), azino-amidine (N^1^,N^1^-dimethyl-N^2^-β-(2-pyridylethyl)formamidine), biguanides (biguanide, metformin, and imeglimin), and nitriles (different series of mono- and di-substituted (cycloprop-2-en-1-ylidene)acetonitrile and (cycloprop-2-en-1-ylidene)cyanamide). The neutral and protonated forms of **Lys**, **Cad**, **AEP**, **FDMEP**, and nitriles display only rotational isomerism, whereas those of the other derivatives—**His**, **Arg**, **HA**, **Agm**, **BG**, **M**, and **I**—exhibit additionally prototropy. All of them contain more than one N atom that are potentially protonated in the gas phase.

The structural analysis for three non-zwitterionic forms of N-containing α-amino acids (**Lys**, **His**, and **Arg**, see Figure 3), was clearly described in 2012 by Bouchoux in his review article for α-amino acids [61]. After analysis of all possible isomers for their neutral and protonated forms, the molar fraction *x*_i_ and *y*_i_ for isomers being in Equilibrium (1) were estimated at 298 K from Equation (12), then PA and GB were calculated according to Equations (13) and (14), where *H*_i_ and *G*_i_ are the enthalpy and Gibbs energy for individual isomer, respectively. Calculations were carried out for the three α-amino acids at the G3MP2B3 and/or G4MP2 levels.

*x*_i_ (or *y*_i_) = exp(−Δ*G*_i_/*RT*)/(∑_1_^n^exp(−Δ*G*_i_/*RT*) (12)

PA(Base) = ∑_1_^n^*x*_i_*H_i_*(Base) + *H*(H^+^) − ∑_1_^n^*y*_i_*H_i_*(conjugate Acid) (13)

GB(Base) = ∑_1_^n^*x*_i_*G_i_*(Base) + *G*(H^+^) − ∑_1_^n^*y*_i_*G_i_*(conjugate Acid) (14)

Bouchoux compared the G*n*-calculated PAs to various experimental and theoretical data and recommended the evaluated PA/GB values for the three amino acids as equal to 994/952, 979/947 and 1046/1007 kJ mol^−1^, respectively. The PA values of **Lys** and **His** are very close to 1000 kJ mol^−1^, whereas that of **Arg** indicates that this molecule already possesses superbasic properties. Note that superbasic cyclic amidines, 1,5-diazabicyclo[4.3.0]non-5-ene (**DBN**) and 1,8-diazabicyclo[5.4.0]undec-7-ene (**DBU**), measured by us in the 1990s, were used by Wu and Fenselau in 1992 as reference bases for the first experimental determination of PA for **Arg** [65]. The first experimental PA/GB values of **Arg** evaluated by Hunter and Lias in 1998 (1051.0/1006.6 kJ mol^−1^ [9]) are close to those evaluated in 2012 by Bouchoux [61]. The favored site of protonation in the gas phase was also proven by theoretical calculations: the 5-amino group in **Lys**, imidazole part (formamidino group) in **His** and the 4-guanidino group in **Arg**. Since the formamidino group in **His** and the guanidino group in **Arg** exhibit tautomerism (the labile proton moves between N atoms), each N atom can take the sp^2^ hybridization in individual tautomers and can be protonated leading to the formamidinium and guanidinium cations, respectively.

Change of conformation when proceeding from neutral to mono-protonated di-amines was also examined in theoretical PA/GB estimations and reviewed in 2012 by Bouchoux and Salpin in another article on poly-functional derivatives [66]. For **Cad**, the following experimental PA/GB were evaluated 1000/946 kJ mol^−1^, and theoretical PA 997 kJ mol^−1^ was computed at the G3MP2B3 level. Chelation of the proton by amino groups in bi- (or poly-) amines, and the formation of intramolecular ionic hydrogen bonds strongly affect thermodynamic gas-phase basicity parameters. These effects were earlier studied by numerous chemists in U.S.A and Canada and additionally reviewed first in 2005 and next in 2012 by Meot-Ner [67,68]. In the case of **Cad**, when compared to n-pentylamine, the PA/GB values increase by 76/57 kJ mol^−1^ according to the evaluation by Hunter and Lias [9].

Isomeric (rotational and/or prototropic) states of neutral and protonated forms and also the favored sites of protonation in the other derivatives given in Figure 3 (**HA**, **Agm**, **AEP**, **FDMEP**, **BG**, **M**, **I**, and nitriles) have been analyzed by us at various DFT and/or MP2 levels over the last 20 years [69,70,71,72,73,74,75]. **For HA**, **AEP**, and **FDMEP**, their GB values were also determined by FT-ICR equilibrium measurements (960.3, 954.8, and 1008.8 kJ mol^−1^, respectively, evaluated in Ref. [18]). They are considerably higher than those predicted on the basis of gas-phase substituent effects, confirming their exceptional gas-phase basicity. By theoretical investigations of **HA**, **Agm**, **AEP**, and **FDMEP** (HF, MP2, and/or DFT), analogous changes in their conformation were confirmed when going from the neutral to protonated forms, such as in di-amines. The formation of intramolecular ionic hydrogen bonds in the conjugate acid forms and exceptionally high gas-phase proton basicity were also validated. For **Agm**, theoretically estimated PA/GB (1062/1019 kJ mol^−1^ at the DFT level) are higher than those evaluated by us for **HA** (998.2/960.3 kJ mol^−1^ [18]). Additionally, tautomeric forms play an important role in the neutral isomeric mixtures of **HA** and **Agm**. The protonation-favored group in the gas phase was also identified by both computations and analyses of experimental gas-phase substituent effects. They are as follows: the imidazole part in **HA** (containing the formamidino group, like in **His**), the 4-guanidino group in **Agm** (like in **Arg**), the pyridino N-sp^2^ atom in **AEP**, and the formamidino N-sp^2^ atom in **FDMEP**.

In the case of DFT-investigated series of mono- and di-substituted nitriles containing the methylenecyclopropene and cyclopropenimine scaffolds intercalated between the electron donor guanidino (N=C(NMe_2_)_2_} and/or phosphazeno {N=P(NMe_2_)_3_) group(s) and the electron acceptor cyano group, the complete analysis of all rotational isomers and the potential protonation sites led to the following conclusions. Although change in conformation of the donor group strongly influences basicity of the potential sites of protonation, the cyano group displays higher basicity than the guanidino and phosphazeno substituents for all investigated derivatives. The PA/GB values calculated according to Equations (12)–(14) are higher for derivatives of cycloprop-2-en-1-ylidene)cyanamide than those of (cycloprop-2-en-1-ylidene)acetonitrile by ca. 10 kJ mol^−1^. The PA values for mono-substituted derivatives are slightly smaller than that of **DMAN** and those for di-substituted compounds are close to those of phosphazenes. They are between 1000 and 1100 kJ mol^−1^.

In the literature, there are numerous theoretical articles in which possible isomeric states have not been considered for the base and conjugate acid forms of strong polyfunctional organic N bases with flexible structures. Thermodynamic parameters were calculated for one neutral and one protonated form, leading to estimation of PA and GB being valid only for the considered base/conjugate acid pair. Such estimations can result in misinterpretations and errors, particularly when selected structures for the acid and base forms have not the lowest Gibbs energies among all possible neutral and protonated isomers. Only cases in which two selected neutral and protonated isomers possess the lowest Gibbs energies, lower than those of the other isomers by more than 20 kJ mol^−1^, can the PA and GB be estimated from the enthalpies and Gibbs energies of the favored low-energy acid and base isomers.

For example, in theoretical investigations of gas-phase proton basicity carried out and reported by the Maksić group and also by the Koppel group for polyimines and polyguanides and additionally for derivatives containing the guanidino and/or phosphazeno substituents in polyfunctional guanidinophosphazenes, polyphosphazenes, guanidinophosphines, phosphazenophosphines, etc., there is no information if isomeric analyses or if low-energy isomers that dictate the calculated PAs were selected (see for example Refs. [17,19,60]). These incomplete literature computational data involve only microscopic value. They only give some information on internal effects in selected isomeric forms. Additional detailed conformational studies for both neutral and protonated species are needed for estimating PAs valid for isomeric systems.

Numerous incomplete theoretical works can also be found in the literature for tautomeric organic push–pull nitrogen bases containing at least one labile (tautomeric) proton in a pushing amino group and a N-sp^2^ atom in a pulling group (C=N or P=N). For the parent compounds (containing unsubstituted amino group(s)), exceptionally rare protomers (estimated mole fraction < 1 ppm) were considered and their PAs/GBs calculated. In conditions where an equilibrium between tautomers is possible (protonation–deprotonation reactions), their participation in the tautomeric mixture is negligible and the calculated PAs/GBs almost useless. Although presenting extremely low stability, structures of some rare tautomers were proposed as possible candidates of superbases for future measurements. Unfortunately, such predictions have only informative value for future experimental studies of their derivatives, for which prototropy is no longer possible.

For example, almost 30 years ago in an article on the basicity of acetamidine (H_2_NC(Me)=NH) [76], its rare tautomeric form ((H_2_N)_2_C=CH_2_, Δ*E*: 42 kJ mol^−1^ at the G2(MP2) level) was proposed as a very strong carbon base with stronger basicity than guanidine ((H_2_N)_2_C=NH). The PA/GB values of this rare form of acetamidine (ca. 5 × 10^−6^% in the tautomeric mixture) is not measurable because of its highly unstable nature. On the other hand, the diaminoalkyl-ethylene groups without possibility of prototropy ((R_2_N)_2_C=C<, R: alkyl) may be considered as strong pushing substituents in various conjugated organic bases containing pulling imino groups. (R_2_N)_2_C=CH_2_ may be also a superbase.

**Figure 4:**
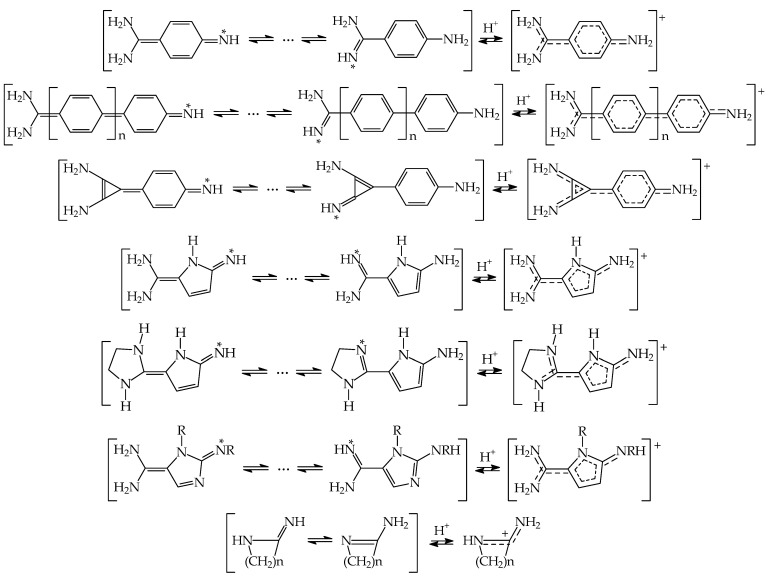
Examples of push–pull imines (structures on the left side of the equilibria) for which prototropy and stabilities of other tautomers (particularly second aromatic structures) were not considered in theoretical PA investigations reported by the Maksić group [60,77,78,79,80], Rouhani et al. [81,82], and Bouchoux and Eckert-Maksić [56]. N-sp^2^ sites of protonation in selected tautomers indicated by an asterisk. Protonation of selected tautomeric pairs leads to common conjugate acid forms.

The (R_2_N)_2_C=C< groups were frequently considered in theoretical search of strong organic nitrogen bases by Maksić and co-workers (see for example Refs. [60,77,78,79,80]), and also by Rouhani et al. (see for example Refs. [81,82]). However, the (H_2_N)_2_C=C< group was also applied without analysis of its possible tautomeric conversion in series of quinonimines, 2,5-dihydropyrrolimines, and 2,5-dihydroxyimidazolimines. This group can participate in prototropy with the pulling imino group, leading to other tautomers. Owing to high aromatic stabilization of the benzene, pyrrole, and imidazole rings, the aromatic forms can dictate the structure and basicity of push–pull imines (see examples in Figure 4). The reported PAs referring to rare tautomers have rather informative value on their so-called “microscopic” basicities. To our knowledge, the stability of the quinonimine form of 4-aminobenzamidine and the exo imino forms of azoles have never been experimentally proven. In the case of azoles, the exo imino forms of 2- and 4(5)-aminoimidazoles have been only theoretically investigated [83]. These protomers (<0.001%) possess considerably weaker electron delocalization and higher Gibbs energies than those of the dominating exo-amino forms.

Even for very simple cyclic tautomeric amidines containing at least one H atom at the amino N atom (last example in Figure 4), Bouchoux and Eckert-Maksić did not analyze prototropy (nor configurational isomerism for C=NH) in their theoretical investigation to indicate the isomeric form(s) that dictate the calculated PA/GB values [56]. Tautomeric equilibria are also possible for some unsymmetrically N,N′,N″-trisubstituted guanidines experimentally and theoretically investigated by Glasovac et al., but there is no information on such types of analyses in the theoretical parts of their works [26,27].

**Figure 5:**
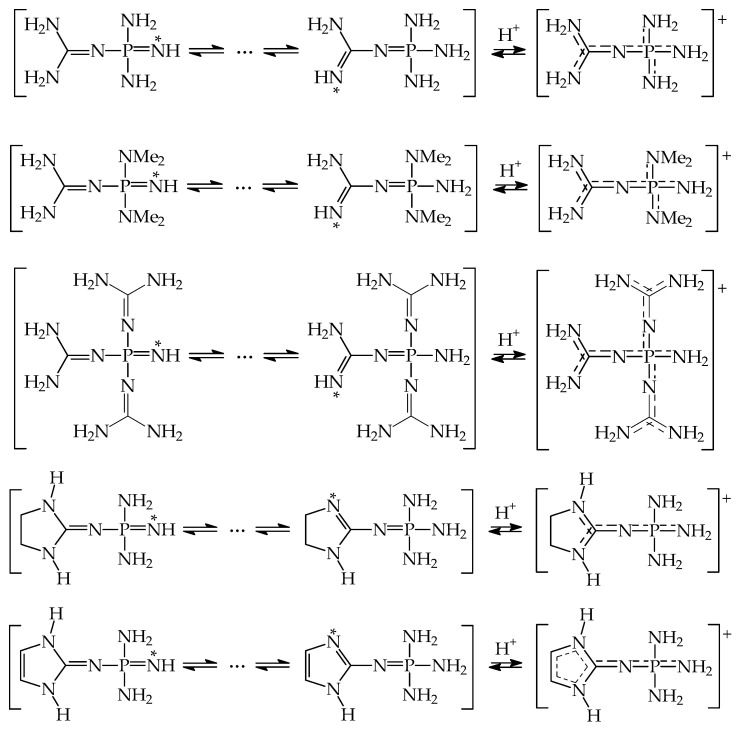
Examples of phosphazenes [84] possessing >NH or NH_2_ group(s) conjugated with P=N for which prototropy was not considered in the literature’s theoretical investigations. N-sp^2^ sites of protonation in selected tautomers indicated by an asterisk. Protonation of selected tautomeric pairs leads to common conjugate acid forms.

Additionally, prototropic conversions can take place for variously substituted guanidinophosphazenes possessing pushing amino NH_2_ or >NH group(s) conjugated with the pulling P=N site. However, this phenomenon has not been considered by Koppel, Leito, Kolomeitsev, et al. in their theoretical work [84] (see examples of phosphimines in Figure 5). Tautomeric equilibria, tautomeric preferences, and basicity of tautomeric mixtures for such type of derivatives will need support from quantum chemical calculations in the future. Note that according to acid–base equilibria for tautomeric systems, less basic tautomers predominate in the tautomeric mixtures and determine the structure and acid–base properties. The labile proton prefers the most basic site in tautomeric moiety. Larger differences between GBs of tautomeric sites involve lower percentage contents of rare tautomer possessing higher basicity (higher GB).

The literature computational data for tautomeric amino-imine (amidines, guanidines, and azoles) and amino-phosphimine superbases (guanidinophosphazenes) and also for their polymeric analogs give only information on microscopic basicity and internal push–pull effects for rare forms. For planning future basicity measurements, the complete structural analyses, and the appropriate estimations of PAs/GBs for dominant protomers–rotamers that could be synthesized are needed.

## 7. Series of Brønsted Organic Nitrogen Superbases with Known Experimental GBs

The gas-phase experimental measurements in the superbasicity range were almost all made by proton transfer equilibria, so we discuss here mostly their basicity in terms of GBs. Most values cited henceforth are taken from the Hunter and Lias compilation [9], except for some specific cases. It should be mentioned that the upper part of the 1984 basicity scale [42] was revised in 1998 [9], this scale being recognized as the universal reference. If a gas-phase basicity larger than that of **DMAN** (GB 995.8 kJ mol^−1^ [9]) is taken as the criterion of superbasicity, numerous subfamilies of very strong organic nitrogen bases can be distinguished as superbases in the gas-phase experimental GB scale. They contain mainly N-sp^3^ or N-sp^2^ as a favored site of protonation. Their basicity measurements have been carried out and thermodynamic parameters determined in the last 50 years in numerous laboratories, first in the USA and Canada and next, also in Europe and Asia. Nevertheless, for bases with only one N-sp^3^, all mono-functional primary, secondary, and tertiary amines have experimental GBs lower than that of **DMAN**. Among them, the highest GB value pertains to Bu^n^_3_N (967.6 kJ mol^−1^).

Even acyclic diamines with flexible structures and possible formation of intramolecular ionic hydrogen bonds in protonated forms, such as H_2_N(CH_2_)_n_NH_2_, Me_2_N(CH_2_)_n_NH_2_, and Me_2_N(CH_2_)_n_NMe_2_ (n: 2, 3, 4, or 6) possess experimental GBs between 912.5 (for H_2_N(CH_2_)_2_NH_2_) and 992.7 kJ mol^−1^ (for Me_2_N(CH_2_)_4_NMe_2_). It should be mentioned here that the difference between entropies of neutral and protonated **DMAN** for Equilibrium (1) (*S*(B) − *S*(BH^+^)) was estimated as equal to zero, because steric effects of two NMe_2_ groups make a change in conformation (rotation) impossible. Hence, the entropy change of Equilibrium (1) for **DMAN** is equal to *S*(H^+^). However, for acyclic diamines the additional entropy contribution is estimated to lie between 20 to 70 J mol^−1^ K^−1^ due to the breaking of the internal hydrogen bond present in the protonated forms, in which many rotational degrees of freedom are frozen. In some cases, this leads to higher PAs for acyclic diamines than for **DMAN** (1028.2 kJ mol^−1^), e.g., for Me_2_N(CH_2_)_3_NMe_2_, Me_2_N(CH_2_)_4_NMe_2_, and Me_2_N(CH_2_)_6_NMe_2_ PAs are equal to 1035.2, 1046.3, and 1035.8 kJ mol^−1^, respectively. For details on the history of PA/GB experimental determinations of polyamines, see the review article of Bouchoux and Salpin [66].

In the 1980s, the literature provided examples of amines with high experimental PAs/GBs, but, surprisingly, they were not included in the last Hunter and Lias compilation. For example, in 1980 Meot-Ner et al. [85] used the tertiary amine (i-Pent)_3_N of known PA value as a reference base for PA measurements of two strong acyclic triamines, H_2_N(CH_2_)_2_NH(CH_2_)_2_NH_2_ (993.7 kJ mol^−1^) and H_2_N(CH_2_)_3_NH(CH_2_)_3_NH_2_ (1035.5 kJ mol^−1^), but the evaluated PA values for the two triamines and reference base are not included in the Hunter and Lias compilation. The PA value of the last triamine seems to be even higher than that of **DMAN**. However, the basicity of the reference base (i-Pent)_3_N applied in Ref. [85] was taken from Aue and Bowers [49] and adjusted to the old PA value of ammonia (866 kJ mol^−1^). Applying a correction using the 1998 proton affinity of the anchor point (PA(NH_3_) 853.6 kJ mol^−1^), gives PA values of the two triamines smaller by 12.4 kJ mol^−1^. The triamines are much stronger bases than ammonia, and the large basicity gap between the reference base NH_3_, and these amines may need a reevaluation. In particular, it can be noticed that the PAs of diamines H_2_N(CH_2_)_3_NH_2_ and H_2_N(CH_2_)_4_NH_2_ (985.3 and 1002.1 kJ mol^−1^, respectively) reported in Ref. [85] relative to those evaluated by Hunter and Lias in 1998 (987.0 and 1005.6 kJ mol^−1^, respectively) are only 1.7 and 3.5 kJ mol^−1^ lower, respectively. Other unevaluated PAs for diamines are listed in the NIST database [10], and more experimental studies are in order to reduce the uncertainty on the basicity of triamines.

The question of PAs/GBs for polyamines was also examined by Reyzer and Brodbelt [86], who experimentally investigated, just before publication of the Hunter and Lias compilation, the gas-phase basicity of series of superbasic acyclic polyamines containing 3, 4, 5, or 6 amino groups and additionally series of cyclic tetra-amines. In series of acyclic and cyclic derivatives, the amino groups are separated by 2 or 3 methylene groups (Table 1), like in tri-amines investigated earlier by Meot-Ner et al. [85]. For GBs measurements using the bracketing method, Reyzer and Brodbelt used the 1997 GB values of reference bases accessible in NIST at that time, which were slightly different (by 0.1–1.0 kJ mol^−1^) than those published by Hunter and Lias in 1998. The Reyzer and Brodbelt experimental GBs for polyamines vary from 966 (for tri-amine H_2_N(CH_2_)_2_NH(CH_2_)_2_NH_2_) to 1021 kJ mol^−1^ (for hexa-amine H_2_N(CH_2_)_2_[NH(CH_2_)_2_]_4_NH_2_ and cyclic tetra-amine with 3 methylene groups). A larger number of amino groups and of methylene groups brings higher GB values. As already mentioned, the PA or GB results obtained by the bracketing method have only a semi-quantitative value. Hence, gas-phase basicities of acyclic and cyclic polyamines should be further quantitatively examined, especially by using the equilibrium method.

More than one decade later, Tian et al. [87] applied the equilibrium method and the 1998 Hunter and Lias PA/GB values of the reference base **DBU** (1047.9/1015.5 kJ mol^−1^) for gas-phase basicity measurements of the acyclic tetra-amine (H_2_NCH_2_CH_2_CH_2_)_3_CNH_2_. Although their experiments have quantitative value, the measured PA/GB values (1071.9/1016.7 kJ mol^−1^) need some correction owing to our new experimental PA/GB values of **DBU** (1050.6/1018.2 kJ mol^−1^ [18]). This correction for the tetra-amine (H_2_NCH_2_CH_2_CH_2_)_3_CNH_2_ is included in Table 1. Note that the difference between entropies of neutral and protonated forms cannot be equal to zero for acyclic polyamines. Their PAs can be considerably higher than that of **DMAN**. This question requires further detailed theoretical analyses of conformational states for the neutral and protonated forms of polyamines. Some proposals for the isomeric states for triamines have been already reported in 1980 by Meot-Ner et al. [85].

Generally, mono-functional imines, containing only N-sp^2^ as a protonation site, are weaker bases than the corresponding amines [9]. The experimental GBs of alkyl- and aryl-substituted imines, aromatic azines and azoles are lower than that of **DMAN**. Even aromatic push–pull azines substituted by one strong electron donating group (NM_2_ or N=CHNMe_2_) display lower experimental GBs than **DMAN** [9,88,89,90]. Only derivatives containing stronger electron-donating groups, such as N=C(NR_2_)_2_ and N=P(NR_2_)_3_, introduced in aromatic mono- and poly-azines can sufficiently increase the GBs of the aza group above 1300 kJ mol^−1^. This was theoretically confirmed by Maksić et al. [60,91].

**Figure 6:**
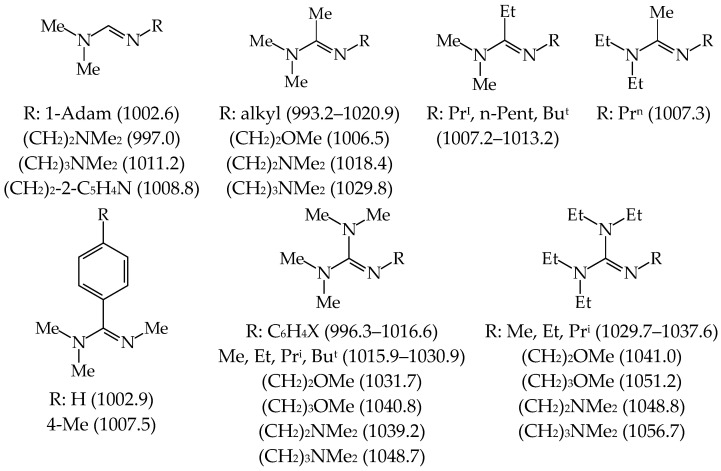
Experimental GBs (in kJ mol^−1^) for superbasic acyclic N^1^,N^1^-dialkyl- and N^1^,N^1^,N^2^-trialkylamidines and N^1^,N^1^,N^3^,N^3^-tetralkyl-guanidines taken from Ref. [18].

In the period of 1990–2004, investigations for series of push–pull N^1^,N^1^-dimethyl-N^2^-substituted formamidines (Me_2_NCH=NR, R: Ph, C_6_H_5_X, CH_2_Ph, CH_2_C≡CH, CH_2_CH=CH_2_, alkyl, cycloalkyl, heteroalkyl, and pyridyloalkyl), were carried out first at the Federal Institute of Technology in Lausanne (Switzerland) for a few aryl derivatives [51], then at the University Nice Sophia Antipolis (now the University Côte d’Azur, France) for all derivatives in both cases using the equilibrium method [52,53,54,55]. Interestingly, investigated formamidine derivatives, such as Me_2_NCH=NR, display considerably stronger gas-phase basicity than the corresponding primary amines, H_2_NR. Moreover, the difference between the GBs of the weakest base (Me_2_NCH=NCH_2_C≡N) and the strongest one (Me_2_NCH=N(CH_2_)_3_NMe_2_) in this series is close to 100 kJ mol^−1^. Changing only one substituent at the N-sp^2^ atom allows us to proceed from derivatives with GBs close to that of medium basicity strength Et_2_NH (919.4 kJ mol^−1^) to those with GBs higher than that of the strong base **DMAN**. Four formamidines from this series can be classified as superbases, Me_2_NCH=N(CH_2_)_2_NMe_2_, Me_2_NCH=N-1-Adam, Me_2_NCH=N(CH_2_)_2_-2-C_5_H_4_N, and Me_2_NCH=N(CH_2_)_3_NMe_2_ with evaluated experimental GB equal to 997.0, 1002.6, 1008.8, and 1011.2 kJ mol^−1^, respectively (Figure 6) [18].

Encouraged by these results, the experimental PA/GB scale was extended by us up to 1100 kJ mol^−1^ by successive equilibrium GB measurements carried out for different series of acyclic amidines (Me_2_NC(Me)=NR, R: alkyl and heteroalkyl; Me_2_NC(Et)=NR, R: alkyl; Et_2_NC(Me)=NR, R: alkyl; and Me_2_NC(C_6_H_4_R)=NMe, R: 4-NO_2_, H, and 4-Me) and guanidines ((Me_2_N)_2_C=NR, R: Ph, C_6_H_4_X, alkyl, heteroalkyl; and (Et_2_N)_2_C=NR, R: alkyl and heteroalkyl) [20,21,22,23]. Figure 6 includes the experimental GBs for all acyclic amidines and guanidines, stronger than **DMAN**. All of them were synthesized and purified by one of us (E.D.R.), like the first series of formamidines. Experimental GB data in Figure 6 are those reported in Ref. [18].

**Figure 7:**
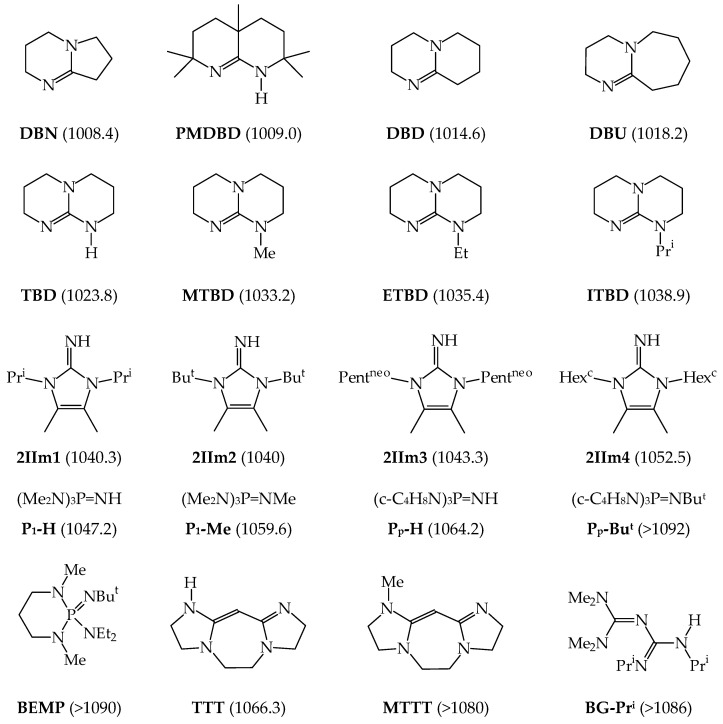
Structures of superbasic cyclic amidines (**DBN**, **PMDBD**, **DBD**, and **DBU**), cyclic guanidines (**TBD**, **MTBD**, **ETBD**, and **ITBD**), 2-iminoimidazolines (**2IIm1**, **2IIm2**, **2IIm3**, and **2IIm4**), phosphazenes (**P_1_-H**, **P_1_-Me**, **P_p_-H**, **P_p_-Bu^t^**, and **BEMP**), vinamidines (**TTT** and **MTTT**), and biguanide (**BG-Pr^i^**), and their experimental GBs (in parentheses, in kJ mol^−1^) taken from Ref. [18].

The GB measurements for acyclic amidines and acyclic guanidines also gave us the possibility to determine the gas-phase basicity for cyclic amidines and cyclic guanidines [20,21,22], already known as strong bases in solution, such as **DBN**, **PMDBD**, **DBD**, **DBU**, **TBD**, **MTBD**, **ETBD**, **ITBD**, and additionally to perform preliminary measurements for five phosphazenes, **P_1_-H**, **P_1_-Me**, **P_p_-H**, **P_p_-Me**, and **BEMP**, and two vinamidines, **TTT** and **MTTT**, synthesized earlier by Schwesinger [92]. GB for one biguanide **BG-i-Pr** [23], synthesized by Gelbard [93], was also experimentally estimated by us. The structures of these derivatives and their experimental GBs are given in Figure 7. In this figure, the structures of four 2-iminoimidazolines (**2IIm1**, **2IIm2**, **2IIm3**, and **2IIm4**) that can be considered as guanidines are also included together with their GBs measured by Schröder et al. using the kinetic method and **DBU**, **TBD**, and **MTBD** as reference bases [94]. Experimental GB data are taken from Ref. [18].

At the turn of the 21st century, Glasovac et al. (Ruđer Bošković Institute Zagreb, Croatia) in cooperation with Schröder (Institute of Organic Chemistry and Biochemistry, Prague, Czech Republic) and Schwarz groups (Technical University, Berlin, Germany) undertook syntheses and basicity measurements in the gas phase for acyclic N,N,N′-trisubstituted guanidines ((R’HN)_2_C=NR, R, R’: alkyl, heteroalkyl, or 2-pyridyloethyl) and bisguanidine ((Pr^i^HN)_2_C=N(CH_2_)_3_N=C(NHPr^i^)_2_) [26,27] using the extended kinetic method [39], or the entropy corrected kinetic method [27]. They also synthesized a series of biguanides ((Me_2_N)_2_C=NC(=NR)NHR’, R: alkyl, heteroalkyl, Ph, 4-C_6_H_4_OMe, R’: alkyl, heteroalkyl) and measured their GBs in cooperation with the Leito group (Tartu University, Tartu, Estonia) using two procedures, the proton transfer equilibrium and the extended kinetic method [28]. The highest GBs (≥1050 kJ mol^−1^) were measured for biguanides, bisguanidine, and polydentate guanidines possessing one, two, or three groups—such as (CH_2_)_3_OMe, (CH_2_)_3_NMe_2_, and (CH_2_)_2_C_5_H_4_N, respectively—able to form hydrogen bonds with the protonated site (=NH^+^–). Other polydentate guanidines with higher GBs were studied only theoretically (see for example Refs. [95,96]). The experimental GBs for N,N,N′-trisubstituted guanidines and bisguanidine are summarized in Table 2. The newest GBs for biguanides are given in Table 3.

**Figure 8:**
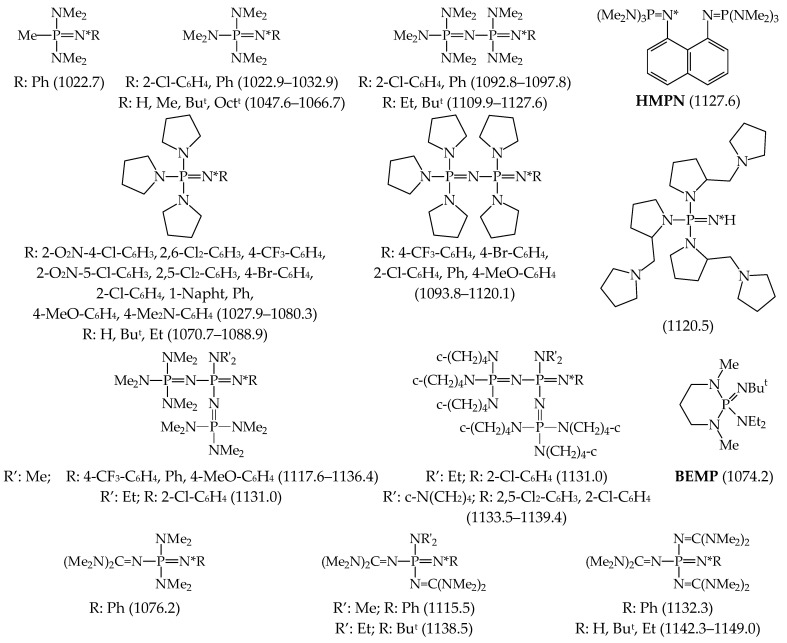
Structures of phosphazenes and their experimental GBs (in parentheses, in kJ mol^−1^) taken from Ref. [18]. The sign * indicates the site of protonation. Abbreviations are listed after Section 10.

About 20 years ago, Koppel, Leito et al. started their experimental GBs measurements using FT-ICR for phosphazenes {R′R″R‴P=NR}, first in cooperation with Mishima at Kyushu University (Fukuoka, Japan) and then in their own laboratory at University of Tartu (Tartu, Estonia) using the equilibrium method in both cases [24,25,57]. Most investigated phosphazenes were prepared by known procedures. Substituent R at the N-sp^2^ atom was varied from H and aryl (1-Napht, Ph, 4-Me_2_N-C_6_H_4_, 4-MeO-C_6_H_4_, 4-Br-C_6_H_4_, 4-CF_3-_C_6_H_4_, 2-Cl-C_6_H_4_, 2,5-Cl_2_-C_6_H_3_, 2,6-Cl_2_-C_6_H_3_, 2-O_2_N-4-Cl-C_6_H_3_, and 2-O_2_N-5-Cl-C_6_H_3_) to alkyl groups (Me, Et, Bu^t^, and Oct^t^), while R’, R”, and R”’ at the P atom were varied from alkyl (Me) and pushing groups (NMe_2_, c-NC_4_H_8_, N=C(NMe_2_)_2_, and N=P(NMe_2_)_3_) to bidentate group (c-NC_4_H_8_-2-CH_2_-c-NC_4_H_8_). The experimental GBs (given in Figure 8, taken from Ref. [18]) are in the range from 1022.7 (for (Me_2_N)_2_P(Me)=NPh) to 1149.0 kJ mol^−1^ (for [(Me_2_N)_2_C=N]_3_P=NEt).

The wide choice among possible substituents at the P and N atoms in phosphazenes generates a large number of possible derivatives. From data in Figure 8, it is logically expected that the progressive substitution by increasingly pushing groups on the P=N-R proton-accepting site will increase the basicity of phosphazenes. Schwesinger et al. introduced the principle of the “battery cell” [97] consisting in the progressive addition in series of more conjugated P=N moieties to the P=NR functional group for increasing the basicity of phosphazenes. Using a computational approach, Koppel, Leito, and their co-workers found that this increase suffers in fact an exponential saturation, with a PA/GB limit of ca. 1400 kJ mol^−1^ for polyphosphazenes and guanidinophosphazenes [17,19]. In comparison, this theoretical PA/GB limit for phosphazenes is close to the experimental PA/GB of the strongest inorganic base Cs_2_O included in the Hunter and Lias scale [9].

**Figure 9:**
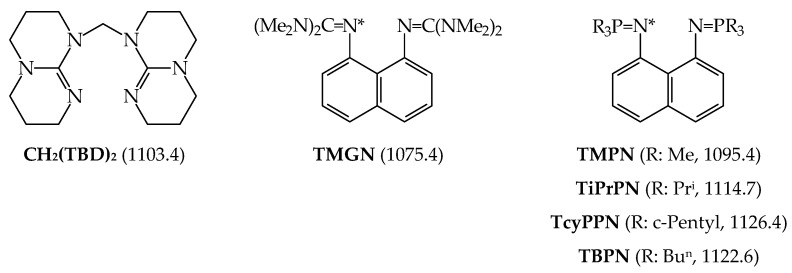
Structures of proton sponge superbases and their experimental GBs (in parentheses, in kJ mol^−1^) taken from Ref. [18]. The sign * indicates the site of protonation. Abbreviations are listed after Section 10.

Koppel and Leito groups also investigated gas phase basicity for series of synthesized superbases on the principle of proton sponges [25]. These molecules contain two N-sp^2^ atoms that can chelate the proton, like **DMAN**. Their GBs were measured using the equilibrium method using phosphazenes as reference bases. The structure of phosphazene derivative of 1,8-diaminonaphthalene (**HMPN**) and its GB is given in Figure 8. The structures of other derivatives from this series of proton sponges (called **TMGN**, **TMPN**, **TiPrPN**, **TcyPPN**, **TBPN**, and **CH_2_(TBD)_2_** by the authors), and their GBs are included in Figure 9. The GBs are taken from Ref. [18].

The third important family of N-bases is nitriles that possess the N-sp atom as the protonation site. Nitriles are intrinsically weaker bases than amines and imines. Mono-functional nitriles display considerably weaker basicity than the corresponding amines and imines. Since substituent variability is possible only at the cyano-C atom, all GBs measured for commercial or synthesized derivatives are considerably lower than that of **DMAN**. They are between 600 and 900 kJ mol^−1^ [9]. The highest experimental GBs, confirmed by quantum chemical computations were reported for push–pull derivatives [98,99]. The experimental GBs of Me_2_N–CH=N–C≡N (857.3 kJ mol^−1^) and Me_2_N–CH=CH–C≡N (864.3 kJ mol^−1^) are higher than that of NH_3_ (819.0 kJ mol^−1^) but close to that of MeNH_2_ (864.5 kJ mol^−1^) [9]. On the other hand, detailed theoretical studies for various series of push–pull nitriles with guanidino and phosphazeno groups linked directly to the cyano-C atom or separated by a resonance transmitter indicated that it is possible to extend the GB scale for nitriles up to 1100 kJ mol^−1^ [74,75,100,101]. The calculated strong electron donating effects of pushing groups may serve as a guide for preparation of nitrile superbases.

## 8. Substituent Effects and Intramolecular Interactions on Superbasicity of N Bases

Substituent effects for organic nitrogen bases in the gas phase have been already analyzed when their PA/GB measurements started [50]. These internal effects are rather different than those in solution. Taft and Topsom, investigating the nature of substituents electronic effects in the gas phase during the 1980s, proposed a model (a linear free energy relationship) for quantitative analysis of these effects [102]. According to this model, the total gas-phase electronic substituent effect on the PA/GB value can be separated into three components: polarizability, field/inductive, and resonance effects. For each component, Taft and co-workers proposed the corresponding substituent constants, *σ*_α_, *σ*_F_, and *σ*_R_, respectively [103] that can be used for quantitative analyses of gas-phase basicity by employing multilinear regression. This procedure has been well accepted and frequently applied in physical organic chemistry, particularly for organic nitrogen bases such as amines, pyridines, amidines, guanidines, phosphazenes, and nitriles [18,25,52,53,54,102,104,105,106]. In the case of polydentate bases, a fourth component of internal effects may be detected [85]. It refers to intramolecular interactions between the proton of the protonated site and the other basic site(s) that increase the PA/GB value.

Resonance effects, although always accompanied by polarizability and field/inductive effects, play a pivotal role in neutral organic nitrogen superbases of the D–A or D–T–A type (D, A, and T are donor, acceptor, and transmitter groups, respectively). Due to the conjugation in these systems, there is an important electron transfer from D to A, featuring the “push-pull” effect by reference to the electron flow. When D is directly linked to A, PAs/GBs strongly increase for the potential protonation site A, and strongly decrease for the potential protonation site D. When A and D are separated by a conjugated π-electron spacer (T), PAs/GBs of the site A also increase, but frequently to a smaller degree [18], with the possible exception of polycyclic aromatic azines or imines [60].

At the beginning of PA/GB measurements, effects of simple pushing (D) groups—such as OR, NR_2_, and sometimes SR (R: H or alkyl)—have been mainly considered. These electron-rich groups display a notable gas-phase basicity via their heteroatoms. Depending on the push–pull force in D–A or D–T–A systems, the favored protonation site can change. D acts as a strong electron-donor substituent when A becomes a protonation site. This takes place for carbonyl derivatives, R’C(=O)OR, R’C(=O)NR_2_, or R’_2_NC(=O)NR_2_ (e.g., carboxylic acids, esters, amides, and ureas), as well as in the nitrogen analogs R’C(=NH)NR_2_ or R’_2_NC(=NH)NR_2_ (amidines and guanidines). The sp^2^ heteroatom (instead of sp^3^) binds preferentially the proton in conjugated D–A systems [107]. The same is true for cyanamides R_2_NC≡N in the gas phase, for which the N-sp atom is protonated instead of N-sp^3^ [9,101].

At the end of the 1980s, a strong pushing effect of the Me_2_NCH=N group on basicity of the C=O group in solution was experimentally observed for the first time in acetyl derivative of N^1^,N^1^-dimethylformamidine, Me_2_NCH=N–C(=O)Me [108]. In a non-polar medium (CCl_4_), hydrogen bonding with methanol was mainly formed at the carbonyl O atom instead of imino N. The strong pushing effects of the Me_2_NCH=N group were later confirmed for series of other derivatives of general formula Me_2_NCH=N–A containing different pulling groups (A: CN, COPh, CSPh, and SO_2_Ph) directly attached to the formamidine N-sp^2^ atom [109], as well as for derivative of cyanoguanidine, (Me_2_NCH=N)_2_C=N–C≡N [110], by studying hydrogen bonding with methanol and 4-fluorophenol in a non-polar medium (CCl_4_). From these results, the amidino and guanidino groups have been considered for superbases design as effective pushing (D) substituents. The phosphazeno group (R_2_N)_3_P=N was employed as pushing substituent by Schwesinger, who, to our knowledge, prepared polyphosphazenes and measured their exceptionally strong basicity in solution for the first time [97].

**Figure 10:**
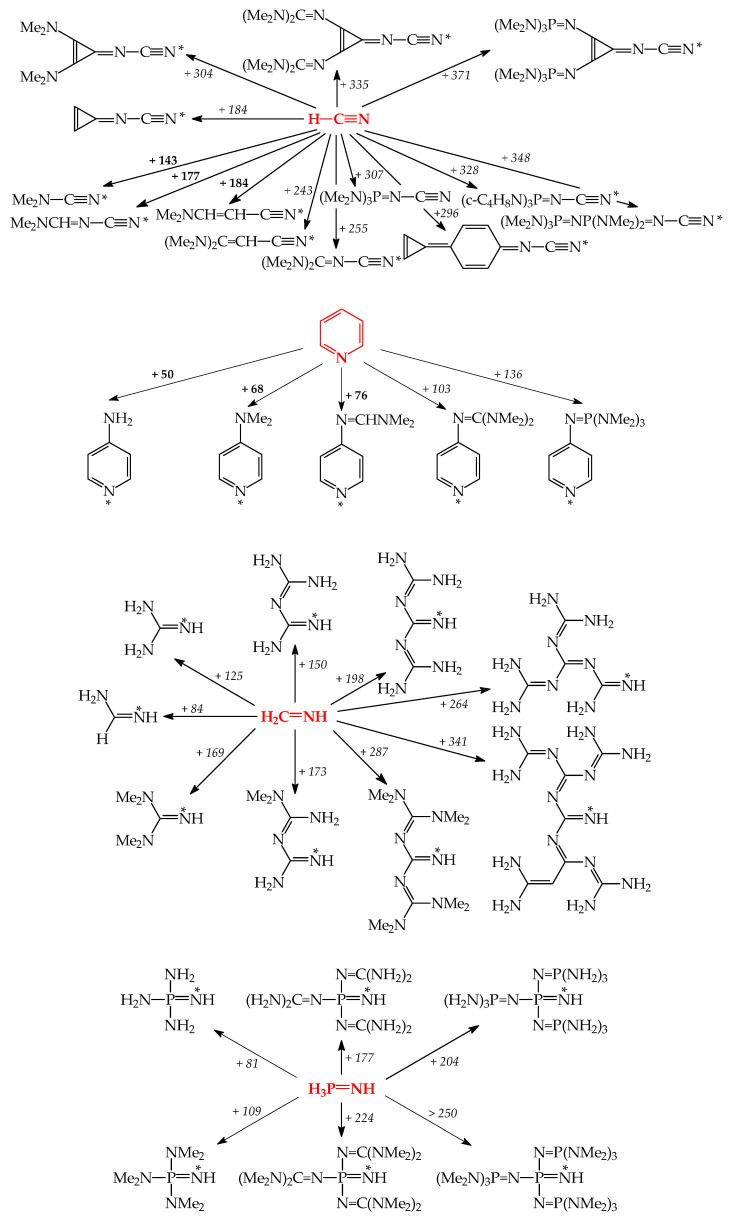
Gas-phase pushing effects (δPA = PA(D–A) − PA(H–A) in kJ mol^−1^) of amino, amidino, guanidino, and phosphazeno groups estimated on the basis of experimental (in bold) [9] or DFT-computed (in italic, mainly microscopic for polyguanides and guanidinophosphazenes) data reported in Refs. [17,60,72,74,75,81,88,89,90,91,99,100,101]. Protonation site in D–A and D–T–A systems indicated by an asterisk, and the parent (unsubstituted) systems are shown in red.

Gas-phase proton basicity for N^2^-cyano-N^1^,N^1^-dimethylformamidine (Me_2_NCH=N–C≡N) was investigated in the Taft’s laboratory [98]. The structure of Me_2_NCH=N–C≡N and the N-sp atom as the site of protonation were additionally confirmed by ab initio calculations [99]. Investigations revealed that the gas-phase pushing effect (defined as δPA = PA(D–C≡N) − PA(H–C≡N)) of the Me_2_NCH=N– group in Me_2_NCH=N–C≡N is stronger than that of Me_2_N– in Me_2_N–C≡N (Figure 10). Then, very strong gas-phase pushing effects of guanidino and phosphazeno substituents, directly attached to the cyano C atom or separated by a π-electron transmitter, were estimated by DFT and/or G*n* computations [74,75,100,101]. In all derivatives, the cyano N-sp atom is the favored site of protonation. When compared to the parent compound, H–C≡N, the guanidino and phosphazeno substituents directly attached to the cyano C atom increase PAs of the cyano N-sp protonation site by more than 250 kJ mol^−1^. Two pushing groups (two NMe_2_, two N=C(NMe_2_)_2_, and two N=P(NMe_2_)_3_) separated by the polarizable and aromatic cyclopropenimino spacer increase PA(C≡N)s by more than 300 kJ mol^−1^ (even for two NMe_2_). However, Y-conjugation of two electron donors in the π-electron spacer significantly attenuates the individual pushing effect in comparison to that of one electron donor directly attached to the pulling C≡N group.

In the case of pyridines, one D substituent at the 4-position, such as NH_2_, NMe_2_, N=CHNMe_2_, N=C(NMe_2_)_2_, and N=P(NMe_2_)_3_ exhibits a sufficient pushing effect so that the endo instead of substituent exo N atoms preferentially binds the proton (Figure 10). This was confirmed by both experimental and computational methods for pyridines with NH_2_, NMe_2_, and N=CHNMe_2_ [9,88,89,105] and only by computations for guanidino and phosphazeno derivatives [91]. When compared to nitriles, the pushing effects of D groups are considerably smaller in pyridines, probably because of separation of D from A by the aryl part. The pushing effects of NMe_2_, N=CHNMe_2_, N=C(NMe_2_)_2_, and N=P(NMe_2_)_3_ in pyridines decrease by ca. 40–50% in comparison to those in nitriles.

When two NR_2_ (NH_2_ or NMe_2_) substituents are directly attached to the imino C atom in guanidines (Figure 10), their pushing effects in Y-conjugation (δPAs of imino N-sp^2^ 125 and 169 kJ mol^−1^, respectively [17]) increase the DFT-computed PAs of the protonation pulling site by more than twice the pushing effects of one NR_2_ in pyridines (δPAs of aza N-sp^2^ 50 and 68 kJ mol^−1^, respectively [9]). On the other hand, Y-conjugation of two NH_2_ groups in guanidine reduces their pushing effects by ca. 74% in comparison to the pushing effect of one NH_2_ group in formamidine. Successive introduction of one guanidino substituent at the imino C atom increases the DFT-computed PAs of the favored N-sp^2^ site of protonation by ca. 150–170 kJ mol^−1^, confirming strong pushing effect of one guanidino group N=C(NR_2_)_2_ or N=C(NR_2_)NR’_2_ in biguanides [72]. Successive introduction of more guanidino N=C(NR_2_)_2_ groups in polyguanides increases the DFT-computed PAs of the favored N-sp^2^ site of protonation by 200–340 kJ mol^−1^ [60,81]. Note that the pushing effects for polyguanides estimated on the basis of incomplete computational data of the Maksić group [60] may refer to microscopic effects.

Although phosphimine (H_3_P=NH) is a stronger base than imine (H_2_C=NH) at the DFT level (Figure 10) [17], three D substituents (NR_2_ and N=C(NR_2_)_2_) increase PAs for phosphazenes to a smaller degree (δPAs ca. 80–110 and 180–220 kJ mol^−1^) than two D substituents for guanidines and diguanidinimines (δPAs ca. 125–170 and 200–290 kJ mol^−1^). It appears that cross-conjugation with the favored N-sp^2^ site of protonation in phosphimine derivatives reduces pushing effects of three D groups to a higher degree than Y-conjugation of two D groups with N-sp^2^ in imine derivatives. This suggests that polyguanides are promising scaffolds for an extension of the PA/GB scale for superbases, stronger than polyphosphazenes.

Substituent polarizability effects of alkyl groups (devoid from resonance effect) also play an important role on gas-phase basicity of neutral organic nitrogen bases. If there are no steric interaction between the substituent(s) and the protonation site, alkyl groups increase gas-phase basicity of the N-sp^3^, N-sp^2^, or N-sp site in the following order: H > Me > Et > Pr^n^ > Pr^i^ > Bu^t^ > 1-Adam. Generally, an increase in the number of C atoms in acyclic or cyclic alkyl group leads to stronger gas-phase basicity of alkyl derivative. For nitriles (RC≡N), only one alkyl group can be substituted at the cyano C atom, and experimental PAs of alkyl derivatives vary from 779.2 for MeC≡N to 834.4 kJ mol^−1^ for 1-AdamC≡N [9]. A comparative study of substituent effects, also including heteroatomic groups, on the basicity of nitriles in the gas phase and in solution demonstrated the importance of the substituent polarizability in the gas phase [111]. In the case of monofunctional amines (RNH_2_, RNHR’, and RR’R”N), one, two, or three alkyl groups can be directly linked to the amino N atom leading to a larger family of N-sp^3^ than N-sp containing bases. The experimental PAs vary from 899.0 for MeNH_2_ to 998.5 kJ mol^−1^ for Bu^n^_3_N [9].

**Figure 11:**
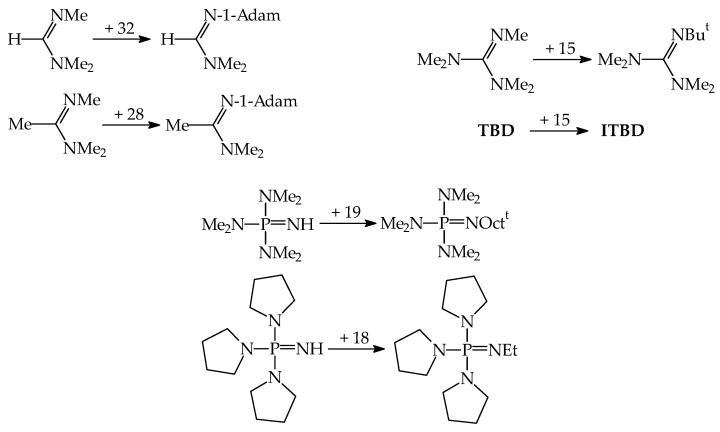
Experimental gas-phase alkyl effects on PAs (in kJ mol^−1^) estimated for selected amidines, guanidines, and phosphazenes [18].

When looking for superbasicity of nitrogen bases, the most interesting scaffolds are imines (RR’C=NR”), phosphimines (RR’R”P=NR”’) and their push–pull derivatives. The possibility of alkyl substitution at N, C, and P atoms considerably increases the number of derivatives in the family of N-sp^2^ containing bases and extends the experimental PA scales from ca. 880 for H_2_C=NMe [112] to 1181.5 kJ mol^−1^ for [(Me_2_N)_2_C=N]_3_P=NEt [18,25]. However, focusing on the substitution at the site of protonation in nitrogen bases, alkyl groups in nitriles and amines act stronger on basicity of the protonation site than in amidines, guanidines, and phosphazenes [9,18]. This suggests that cross-conjugation of amino groups with the protonation N-sp^2^ site in amidines, guanidines, and phosphazenes mitigates the effect of alkyl R group linked to the site of protonation; the stronger the pushing groups, the weaker the R effects. Figure 11 shows some examples of gas-phase alkyl-effects in amidines, guanidines, and phosphazenes. Estimations are based on the experimental PAs/GBs given in Ref. [18].

**Figure 12:**
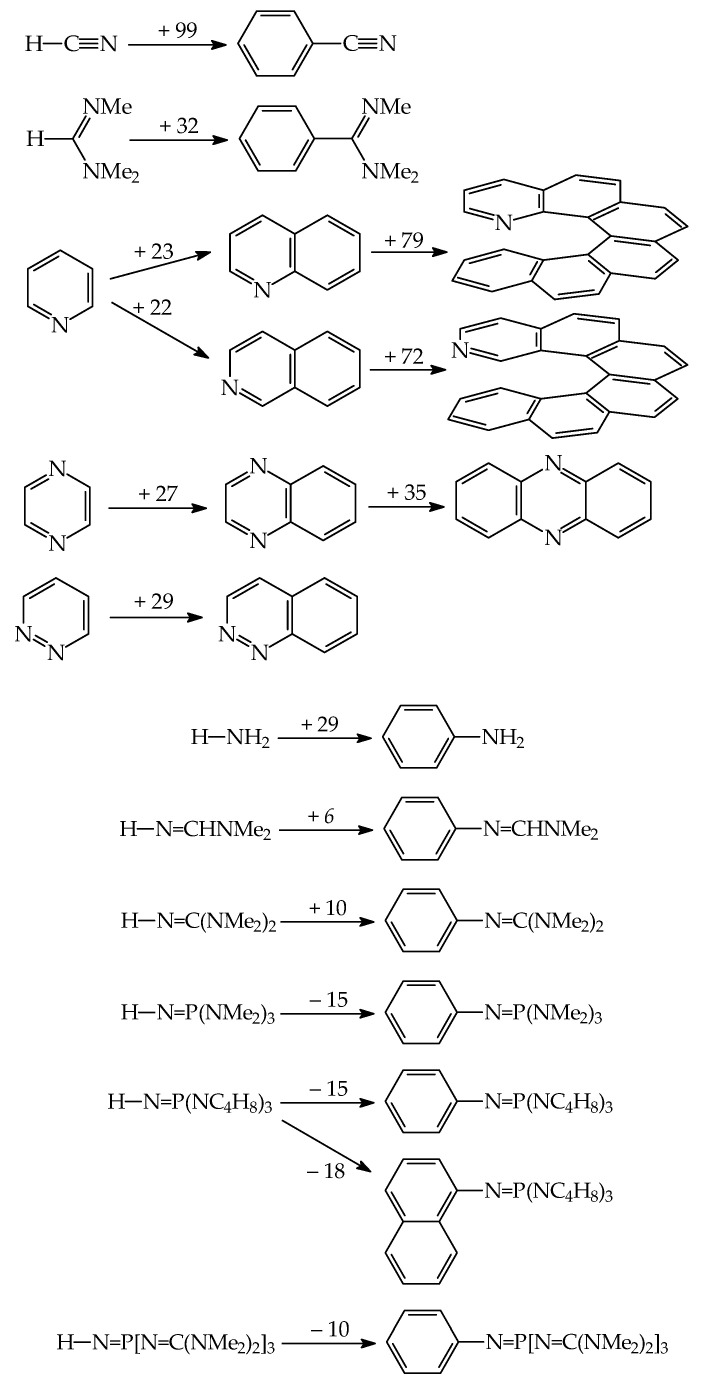
Gas-phase substituent effects and ring fusion on experimental PAs of organic nitrogen bases, from data reported in Refs. [9,18,25,113].

Aryl groups are also promising substituents for superbase design. They can significantly increase the gas-phase basicity of organic nitrogen bases by their exceptional polarizability effects, and also by their resonance effects as potentially electron-donating groups. However, these effects depend on the position and type of derivative (Figure 12) [9,18]. The effect of fusion of additional benzene ring is also favorable for aromatic azines [9,90,113,114,115]. When an aryl group is attached to the site of protonation, N-sp^3^ in amines and N-sp^2^ in amidines and guanidines, the Ph group increases experimental PAs (Figure 12) but to a considerably smaller degree for amidines and guanidines [18] than for aniline [9]. A different situation is observed for monophosphazenes and triguanidinophosphazenes. The aryl groups (Ph and 1-Napht) decrease the experimental PAs in slightly higher degree for monophosphazenes than for triguanidinophosphazenes and also to a slightly higher degree for 1-Napht than Ph [25]. Mono- and poly-cyclic aryl groups, unsubstituted and containing electron-donating substituents, such as NR_2_ (R: H or alkyl), also increase the gas-phase basicity. These interesting effects were described for theoretically investigated derivatives of imines [60,116].

**Figure 13:**
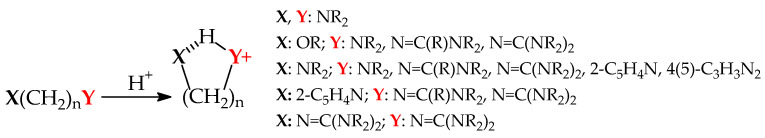
Formation of ionic hydrogen bond in gaseous protonated bidentate nitrogen organic bases. Protonation site indicated in bold red.

Other substituents, such as –(CH_2_)_n_X with π-electrons and/or heteroatom(s) (X: H_2_C=CH, HC≡C, F, NO_2_, CN, CF_3_, CCl_3_, Cl, COMe, etc.), separated from the N-sp^3^, N-sp^2^, or N-sp atom in neutral organic nitrogen bases by one or more methylene groups act as electron-withdrawing groups for which unfavorable (acceptor) X effects are usually higher than favorable (donor) effects of the methylene group(s) [9,18,54]. Generally, the total electronic effect of these substituents decreases the PA/GB values and may be used to adapt the basicity in the design of organic nitrogen superbases. On the other hand, noticeable exceptions are longer and flexible heteroalkyl groups—such as –(CH_2_)_n_OR, –(CH_2_)_n_NR_2_, –(CH_2_)_n_-2-C_5_H_4_N, –(CH_2_)_n_N=C(NR_2_)_2_, etc. (R: H or alkyl, n: 2, 3, 4, or 6)—which can form intramolecular hydrogen bonds between free heteroatoms (O or N) and the protonated site (Figure 13). This internal effect, also called internal solvation, is considerably stronger than the total electronic effects of these heteroalkyl groups. Consequently, their derivatives display PAs higher than those of the corresponding alkyl derivatives, in some cases even by ca. 30 kJ mol^−1^ [18]. Polydentate amines, amidines, and guanidines are the strongest bases in the investigated series of superbases [9,18,20,21,22,23,26,27,54,55,56,60,66,95,96]. Internal solvation also dictates the exceptionally high gas-phase basicity of bidentate α-amino acids (**Lys**, **His**, and **Arg**) [61], their bioamines (**Cad**, **HA**, and **Agm**) [66,69,70] and also 2-(β-aminoethyl)-pyridine (**AEP**) [71] and its derivative N^1^,N^1^-dimethyl-N^2^-β-(2-pyridylethyl)formamidine (**FDMEP**) [55].

In solution, internal solvation effects observed for polydentate nitrogen bases in the gas phase (hydrogen bonding between the protonated and the free nitrogen base) are usually reduced by a competitive external solvation. The intensity of the reduction depends on solvent polarity, ionizing power, and hydrogen bonding ability. Generally, internal solvation is completely eliminated in aqueous solution. Some exceptions can be proton sponges (e.g., **DMAN**). Additionally, other internal (structural) effects such as polarizability, field/inductive and resonance effects are also attenuated to varying degrees by external solvation. Therefore, relations between thermodynamic acidity-basicity parameters in the gas phase (PA/GB) and solution (p*K*_a_) are limited to series of similar bases, and only some local relation for particular subfamilies may be observed. This has been discussed by us in our earlier articles for series of some strong push–pull N-sp^2^ bases, such as amidines [52,54] and guanidines [21], as well as by Koppel et al. for series of phosphazenes [24,25]. Taft et al. analyzed earlier solvent effects for series of aromatic N-sp^2^ bases, pyridines [105].

## 9. Selected Methods for Organic Nitrogen Superbases Preparation

Various methods for the preparation of amidines and guanidines were reviewed in chapters of Patai books [117,118,119]. Amidines can be synthesized from different precursors such as nitriles, amides, thioamides, lactams, orthoesters, amidoximes, keteneimines, carbodiimides, isocyanides, Schiff bases, etc. Guanidines can be prepared from ureas, thioureas, cyanamides, cyanoguanidine, etc. Series of N^1^,N^1^-dialkyl N^2^-substituted amidines, investigated in the gas phase, were prepared by one of us (E.D.R.) [20,21,22,51,52,53,54,55] using the procedure described by the Scoggins’s Reaction (15) [120], in which a mixture of dialkylacetal of N,N-dialkylamide and the corresponding primary amine (1:1) is heated without solvent during 10–20 min. In this reaction, the only side product is MeOH. The dialkylacetal of N,N-dialkylamide was commercial or prepared at low temperature from the reaction of N,N-dialkylamide with MeONa, directly obtained from MeOH and Na [121]. Benzamidines substituted at the phenyl ring and at the N-atoms [20,21] were synthesized by one of us (E.D.R.) from a mixture of iminochloride (obtained from N-substituted benzamide and PCl_5_) and the corresponding primary amine in benzene as solvent according to the well-known Reaction (16) [117]. Unfortunately, this reaction led also to various side-products, and its yield was not very high but was sufficient for isolation and basicity measurements. Prior to FT-ICR experiments, liquid amidines were purified by preparative gas chromatography and solid derivatives by sublimation.
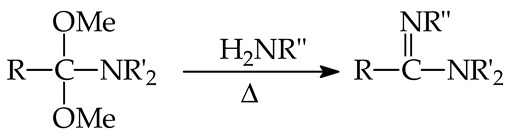
(15)

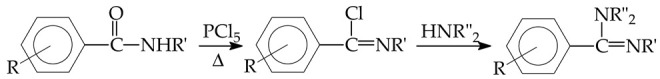
(16)


An analogous procedure (Reaction (17)) was applied by one of us (E.D.R.) for the syntheses of N^1^,N^1^,N^3^,N^3^-tetralkyl-N^2^-substituted guanidines [20,21,22]. In this method, N,N,N′,N′-tetralkylurea was mixed with POCl_3_, then, the primary amine was added [122,123,124]. Tautomeric N^1^,N^2^,N^3^-trialkylguanidines studied by Glasovac et al. [26,27] were mainly prepared according to the known Reaction (18) from disubstituted carbodiimide and primary amine [119].
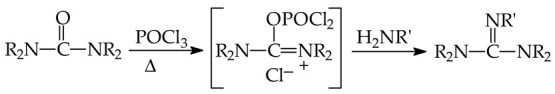
(17)

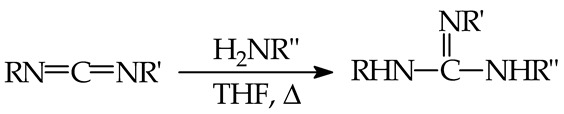
(18)

Different methods for the preparation of biguanides were recently reviewed by Ronco et al. [125]. For example, various biguanides can be prepared from unsubstituted or substituted cyanoguanidine in reaction with primary or secondary amines. They can be also obtained in reaction of disubstituted carbodiimides with unsubstituted or substituted guanidines. Unsubstituted guanidine in reaction with substituted thioureas or cyanamides can be transformed into biguanides. Reaction of sodium dicyanamide with primary or secondary amines can also lead to biguanides. For syntheses of tautomeric hexasubstituted biguanides, Glasovac et al. applied Reaction (19) and used disubstituted carbodiimides and **TMG** as reactants [126]. They also tested different conditions for syntheses and found that the highest yields were obtained using only MW and heating.
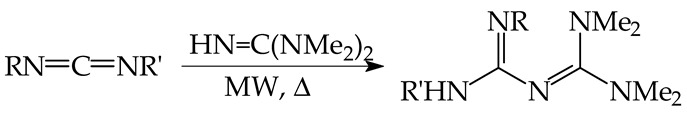
(19)

**Figure 14:**
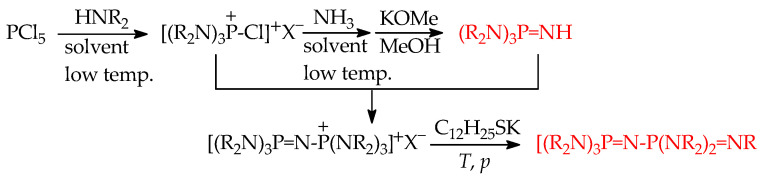
Reaction scheme for preparation of mono- and di-phosphazenes [97]. Phosphazene products are shown in red.

Preparative methods for differently substituted acyclic and cyclic phosphazenes, and polyphosphazenes were developed by Schwesinger et al. [92,97,127,128,129,130,131]. According to the procedure proposed by Schwesinger [97], substituted triaminoalkylphosphazenes can be prepared from PCl_5_ and the corresponding amine according to the reaction given in Figure 14. These phosphazenes can be used for syntheses of diphosphazenes, etc. An analogous procedure was applied by Koppel et al. for the preparation of guanidinophosphazenes. In the first step, HN=C(NMe_2_)_2_ (**TMG**) was added to PCl_5_ instead of amine [84].

Schwesinger [132] also proposed methods of syntheses for very strong polycyclic bases with a vinamidine framework. For example, an unsubstituted vinamidine (**TTT**) can be prepared in reaction of triethylenetetramine with malonodinitrile in the presence of NH_4_Cl, then the free base isolated by reaction with MeONa. Considering the strong basicity of **TTT**, Schwesinger additionally prepared exceptionally strong proton sponges with a vinamidine structure [133].

## 10. Overview and Prospects

Neutral molecules bearing a sp^2^ or sp^3^ nitrogen atom (imino or amino center) as the protonation site are generally strong bases in the gas phase. With the possibility of conjugation of the imino or cyano functional group with n- and/or π-electron donor groups, leading to push–pull systems, the gas-phase basicity can be increased up to high levels. In the current NIST gas-phase basicity compilation [10], the strongest organic bases are centered on N-sp^2^ of the amidino functional group (–C(N<)=N–). In fact, this upper part of the scale should be updated with many experimental data obtained in the last two decades and discussed here. An examination of the literature of this period [18,24,25,26,27,28] reveals the continuous progress made in the domain of experimental gas-phase basicity of superbases overcoming the difficulties encountered: synthesis, isolation, and purification of the potential superbases, availability of the appropriate reference bases, and access to FT-ICR instruments adapted to gas-phase proton transfer experiments. Currently, the experimental GBs of push–pull nitrogen bases, such as guanidines, vinamidines, biguanides and phosphazenes, reached the 1100–1200 kJ mol^−1^ range.

In the same period, major advancements in the understanding of the structure and energetics of protonated molecules were made using quantum chemical calculations. The sites of protonation in molecules containing several nitrogen atoms as potential basic center have been confirmed or identified. With advances in computational tools, the theoretical pursuit for super- and hyperbases led to an explosion of papers. In their 2012 review [60], Maksić, Kovačević, and Vianello gave an account of this remarkable progress, and the search of new super/hyperbases continued to flourish. In our previous review [18], we also cited some references relevant to computational studies of push–pull nitrogen bases. These conjugated systems continue to arouse interest as a canonical structure for strong bases design. In this regard, a few significant papers published after 2016 can be cited [70,72,73,74,75,82,83,116,134,135,136,137,138,139,140,141]. Note that theoretical investigations have no such difficulties as those connected with experiments. Quantum-chemical computations are easier, faster, and cheaper to perform than experiments without such limits as availability, stability, and volatility of compounds.

After the era of intense fundamental search for very strong neutral organic bases, both experimental and theoretical, the current focus is more orientated toward practical problems in relation to organocatalysis. Push–pull nitrogen superbases that are commercially available may be added to the collection of organocatalysts [7,142]. For a broader application in organic synthesis, superbase choice or design should be a compromise between moderate molecular weight, stability in usual reaction conditions, and availability [143].

Indeed, after the fundamental advances in the exploration of superbasicity, a new cycle oriented toward more applied topics is emerging. A few examples of the latest research article or reviews are cited here. Superbase catalysis is one of the rapidly developing applications. One interesting approach to overcome some practical difficulties associated with superbases utilization is the in-situ generation of these catalysts [144]. The availability of chiral superbases is a notable addition to the arsenal of the superbase catalysis for enantioselective synthesis [145]. Another facet of modern organocatalysis is the development of polyfunctional catalysts. In this regard, the presence of a superbasic site in the catalyst is often required [146,147,148,149,150]. In addition to catalysis, recent applications in the field of material science [151,152,153], and for carbon dioxide capture and activation [154,155,156,157,158,159,160], seem to be promising.

**Figure 15:**
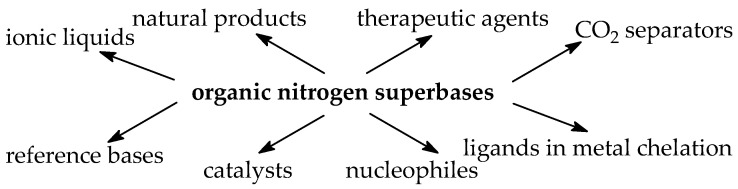
Selected applications of strong organic nitrogen bases.

Many more general uses of molecules acting as superbases may be cited. Amidines, guanidines and biguanides are well recognized since more than 50 years as very important biologically active products such as tranquillizing, antihypertensive, antidiabetics, and anticancer therapeutic agents [161,162,163,164,165,166]. They display antibacterial, antifungal, antiviral, antiprotozoal, and antibiotic activities. Numerous natural products contain the amidino and guanidino groups [167,168]. Strong organic nitrogen bases are also used as ionic liquids [169,170,171,172], ligands in metal chelation [173], and reference bases in PA/GB measurements [18,20,21,22,23,24,25,26,27,28,51,52,53,54,55,65,85,86,87]. Figure 15 summarizes the most significant fields in which organic nitrogen superbases are found.

## Figures and Tables

**Table 1:** Experimental GBs of polyamines compared to that of **DMAN** ^a^.

Structure	GB [Ref.]	Structure	GB [Ref.]
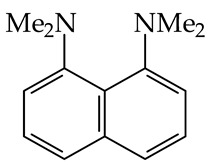		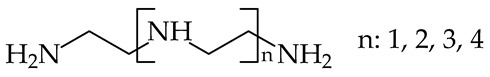	966–1021 [86]
	995.8 [9]	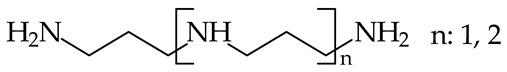	994–1012 [86]
**DMAN**			
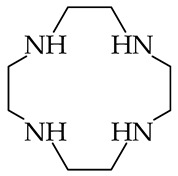		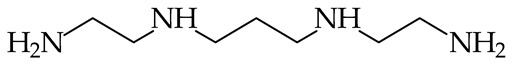	1000 [86]
	1004 [86]	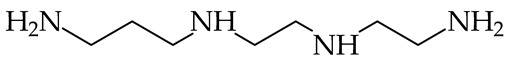	1004 [86]
		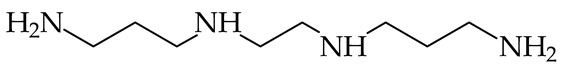	1004 [86]
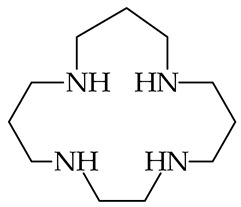	1021 [86]	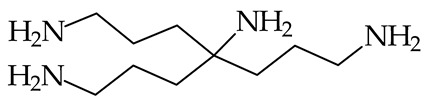	1019 ^b^ [87]

^a^ Experimental error ±8 and ±9 kJ mol^−1^ for derivatives reported in Refs. [86] and [87], respectively. A GB range is given when different structures are shown. ^b^ Corrected according to GB (**DBU**) taken from Ref. [18].

**Table 2:** Experimental GBs (in kJ mol^−1^) for N,N,N′-trisubstituted guanidines and bisguanidine determined by the kinetic method (taken from Ref. [18]).

Derivative	GB	Derivative	GB
(Pr^n^NH)_2_C=NPr^n^	1024	(Pr^n^NH)_2_C=N(CH_2_)_3_NMe_2_	1054
(Pr^i^NH)_2_C=N(CH_2_)_3_SMe	1039	(Pr^i^NH)_2_C=N(CH_2_)_3_NMe_2_	1055
(Me_2_N)_2_C=N(CH_2_)_3_OMe	1050	(MeNH)_2_C=N(CH_2_)_2_-2-C_5_H_4_N	1044
[MeO(CH_2_)_3_NH]_2_C=NPr^n^	1059	(Pr^i^NH)_2_C=N(CH_2_)_2_-2-C_5_H_4_N	1052
(Pr^n^NH)_2_C=N(CH_2_)_3_OMe	1046	[2-C_5_H_4_N-(CH_2_)_2_NH]_2_C=NPr^i^	1068
[MeO(CH_2_)_3_NH]_2_C=N(CH_2_)_3_OMe	1070	[2-C_5_H_4_N-(CH_2_)_2_NH]_2_C=N(CH_2_)_2_-2-C_5_H_4_N	1082
[Me_2_N(CH_2_)_3_NH]_2_C=NPr^n^	1072	(Pr^i^NH)_2_C=N(CH_2_)_3_N=C(NHPr^i^)_2_	1070

**Table 3:** Experimental GBs (in kJ mol^−1^) for biguanides determined by two methods: proton transfer equilibrium (GB_eq_) and kinetic (GB_kin_), taken from Ref. [28]. Protonation sites are indicated by an asterisk. The published values were corrected by +3 kJ mol^−1^, on account of the correction of the NIST values proposed in [18].

Derivative	GB_eq_	GB_kin_
(Me_2_N)_2_C=NC(=N*Ph)NH(CH_2_)_3_NMe_2_	-	1062
(Me_2_N)_2_C=NC(=N*4-C_6_H_4_OMe)NH(CH_2_)_3_NMe_2_	-	1071
(Me_2_N)_2_C=NC(=N*Et)NH(CH_2_)_3_NMe_2_	1089	1085
(Me_2_N)_2_C=NC(=N*Pr^i^)NHPr^i^	1070	1064
(Me_2_N)_2_C=NC(=N*c-C_6_H_11_)NHc-C_6_H_11_	1080	1071
(Me_2_N)_2_C=NC{=N*(CH_2_)_3_OMe}NH(CH_2_)_3_OMe	1095	-
(Me_2_N)_2_C=NC{=N*(CH_2_)_3_NMe_2_}NH(CH_2_)_3_NMe_2_	1106	-

## Data Availability

Not applicable.

## Abbreviations

A	acceptor
A^−^	conjugate base of AH
1-Adam	1-adamantyl group
**AEP**	2-(β-aminoethyl)pyridine
AH	neutral acid
**Arg**	arginine
**Agm**	agmatine (N-(4-aminobutyl)guanidine)
B	neutral base
Bu^n^	n-butyl group
Bu^t^	*tert*-butyl group
**BEMP**	2-*tert*-butylimino-2-diethylamino-1,3-dimethylperhydro-1,3,2-diazaphosporine
BH^+^	conjugate acid of B
**BG**	biguanide (guanidinoamidine)
**Cad**	cadaverine (1,5-diaminopentan)
CCSD(T)	coupled cluster method including single-, double- and triple-excitation
**D**	donor
**DBD**	1,5-diazabicyclo[4.4.0]dec-5-ene
**DBN**	1,5-diazabicyclo[4.3.0]non-5-ene
**DBU**	1,8-diazabicyclo[5.4.0]undec-7-ene
DE	deprotonation enthalpy
DFT	density functional theory
**DMAN**	1,8-bis(dimethylamino)naphthalene
**DMAP**	*N,N-*dimethyl-4-aminopyridine
*E*	energy
*E*_prot_	protonation energy
Et	ethyl group
**ETBD**	7-ethyl-1,5,7-triazabicyclo[4.4.0]dec-5-ene
**FDMEP**	N^1^,N^1^-dimethyl-N^2^-β-(2-pyridylethyl)formamidine
FT-ICR	Fourier transform mode of ICR
*G*	Gibbs energy
G2	Gaussian-2 theory
G2MP2	MP2 variant of Gaussian-2 theory
G3MP2B3	Gaussian-3 theory with B3LYP structures and frequencies, and MP2 energy correction
G4MP2	MP2 variant of Gaussian-4 theory
G*n*	Gaussian-*n* theory
GA	gas-phase acidity
GB	gas-phase basicity
*H*	enthalpy
_f_ *H*	enthalpy of formation
**HA**	histamine
HF	Hartree–Fock self-consistent field
**His**	histidine
**HMPN**	1,8-bis(hexamethylaminophosphazeno)naphthalene
HPMS	high pressure mass spectrometry
*I*	intensity
**I**	imeglimin
ICR	ion cyclotron resonance
**2IIm1**	1,3-di(*iso-*propyl)-4,5-dimethyl-2-iminoimidazoline
**2IIm2**	1,3-di(*tert*-butyl)- 4,5-dimethyl-2-iminoimidazoline
**2IIm3**	1,3-di(*neo*-pentyl)- 4,5-dimethyl-2-iminoimidazoline
**2IIm4**	1,3-dicyclohexyl-4,5-dimethyl-2-iminoimidazoline
**ITBD**	7-*iso*-propyl-1,5,7-triazabicyclo[4.4.0]dec-5-ene
IUPAC	International Union of Pure and Applied Chemistry
*K*	equilibrium constant
**Lys**	lysine
**M**	metformin
Me	methyl group
MP2	second-order Møller–Plesset (perturbation theory)
MP*n*	*n*-order Møller–Plesset (perturbation theory)
**MTBD**	7-methyl-1,5,7-triazabicyclo[4.4.0]dec-7,9-ene
**MTTT**	11-methyl-1,4,7,11-tetraazatricyclo[8.3.0.0^4.8^]tridec-7,9-diene
MW	microwave
NIST	National Institute of Standards and Technology
Oct^t^	*tert*-octyl group
*p*	pressure
**P_1_-H**	tris(dimethylamino)iminophosphorane
**P_1_-Me**	tris(dimethylamino)(methylimino)phosphorane
**P_p_-H**	tris(pyrrolidin-1-yl)iminophosphorane
**P_p_-Bu^t^**	tris(pyrrolidin-1-yl)(tert-butylimino)phosphorane
PA	proton affinity
Ph	phenyl group
p*K*_a_	negative logarithm of acidity dissociation constant
**PMDBD**	3,3,6,9,9-pentamethyl-2,10-diazabicyclo[4.4.0]dec-1-ene
Pr^n^	n-propyl group
Pi^i^	*iso*-propyl group
QCISD(T)	quadratic configuration interaction method including single-, double- and triple-excitation
*R*	molar gas constant
*S*	entropy
*σ_α_*	polarizability substituent constant
*σ_F_*	field/inductive substituent constant
*σ_R_*	resonance substituent constant
*T*	temperature
T	transmitter
**TBD**	1,5,7-triazabicyclo[4.4.0]dec-7,9-ene
**TBPN**	1,8-bis(tri-n-butylphosphoimino)naphthalene
**TcyPPN**	1,8-bis(tri-cyclopentylphosphoimino)naphthalene
**TiPrPN**	1,8-bis(tri-*iso*-propylmethylphosphoimino)naphthalene
**TMG**	N^1^,N^1^,N^3^,N^3^-tetramethylguanidine
**TMGN**	1,8-bis(tetramethylguanidino)naphthalene
**TMPN**	1,8-bis(trimethylphosphoimino)naphthalene
**TTT**	1,4,7,11-tetraazatricyclo[8.3.0.0^4.8^]tridec-7,9-diene
*V*	volume
W1	Weizmann-1 theory
W2	Weizmann-2 theory
*x*	molar fraction of B isomer
*y*	molar fraction of BH^+^ isomer
ZPE	zero-point energy
